# Identification of key amino acid residues toward improving the catalytic activity and substrate specificity of plant-derived cytochrome P450 monooxygenases CYP716A subfamily enzyme for triterpenoid production in *Saccharomyces cerevisiae*

**DOI:** 10.3389/fbioe.2022.955650

**Published:** 2022-08-19

**Authors:** Jutapat Romsuk, Shuhei Yasumoto, Hikaru Seki, Ery Odette Fukushima, Toshiya Muranaka

**Affiliations:** ^1^ Department of Biotechnology, Graduate School of Engineering, Osaka University, Osaka, Japan; ^2^ Industrial Biotechnology Initiative Division, Institute for Open and Transdisciplinary Research Initiatives, Osaka University, Osaka, Japan; ^3^ Plant Traslational Research Group, Universidad Regional Amazónica IKIAM, Tena, Ecuador

**Keywords:** triterpene oxidase, protein engineering, bioinformatics, *in vivo* functional analysis, cytochrome P450 monooxygenase, CYP716A

## Abstract

Triterpenoids constitute a group of specialized plant metabolites with wide structural diversity and high therapeutic value for human health. Cytochrome P450 monooxygenases (CYP) are a family of enzymes important for generating the structural diversity of triterpenoids by catalyzing the site-specific oxidization of the triterpene backbone. The CYP716 enzyme family has been isolated from various plant families as triterpenoid oxidases; however, their experimental crystal structures are not yet available and the detailed catalytic mechanism remains elusive. Here, we address this challenge by integrating bioinformatics approaches with data from other CYP families. *Medicago truncatula* CYP716A12, the first functionally characterized CYP716A subfamily enzyme, was chosen as the model for this study. We performed homology modeling, structural alignment, *in silico* site-directed mutagenesis, and molecular docking analysis to search and screen key amino acid residues relevant to the catalytic activity and substrate specificity of the CYP716A subfamily enzyme in triterpenoid biosynthesis. An *in vivo* functional analysis using engineered yeast that endogenously produced plant-derived triterpenes was performed to elucidate the results. When the amino acids in the signature region and substrate recognition sites (SRSs) were substituted, the product profile of CYP716A12 was modified. We identified amino acid residues that control the substrate contraction of the enzyme (D292) and engineered the enzyme to improve its catalytic activity and substrate specificity (D122, I212, and Q358) for triterpenoid biosynthesis. In addition, we demonstrated the versatility of this strategy by changing the properties of key residues in SRSs to improve the catalytic activity of *Arabidopsis thaliana* CYP716A1 (S356) and CYP716A2 (M206, F210) at C-28 on the triterpene backbone. This research has the potential to help in the production of desired triterpenoids in engineered yeast by increasing the catalytic activity and substrate specificity of plant CYP716A subfamily enzymes.

## Introduction

Triterpenoids are a group of plant specialized metabolites that include diverse bioactive compounds with pharmacological properties, such as anti-cancer, anti-inflammation, anti-viral, anti-diabetes, and anti-microbial activity (Yadav et al., 2010; Yan et al., 2014; Jin et al., 2019). Nevertheless, their production in plants is very low and their purification and chemical synthesis are challenging. Further, the complete biosynthetic pathway has not yet been elucidated in most species. As an alternative method of triterpenoid production, a synthetic biology technique involving expression in heterologous hosts, such as yeast *S. cerevisiae,* has been investigated (Fukushima et al., 2011; Suzuki et al., 2018; Dale et al., 2020). Triterpenoids are synthesized from 2,3-oxidosqualene (a common C-30 precursor that yeast naturally produces) *via* cyclization and site-specific oxidation reactions, catalyzed by oxidosqualene cyclase (OSC) and cytochrome P450 monooxygenases (CYPs), respectively. CYPs are monooxygenases that carry heme as a co-factor and play an important role in diversifying triterpenoid structures by site-specific oxidation of the cyclized backbone (Nelson and Werck-Reichhart, 2011; Fukushima et al., 2013; Seki et al., 2015). In plants, almost all CYPs are connected to the endoplasmic reticulum or chloroplast *via* a membrane anchor followed by a hinge domain. Each CYP contains domains that facilitate interactions with the heme cofactor, a cytochrome P450 reductase domain, and recognition sites that interact with a diverse array of substrates. The heme-binding domain is the most conserved region in the CYP enzyme family because it contains the cysteine ligand and is strong enough to hold the heme in place during oxygenation (Fontana et al., 2005; Sirim et al., 2010; Bak et al., 2011; Sugimoto and Shiro, 2012; Poulos and Johnson, 2015; Prall et al., 2016). The ExxR domain locks the heme pocket into the ligand-binding site and stabilizes the core structure (Poulos and Johnson, 2015; Prall et al., 2016). The PERF domain is required for protein-protein interactions with cytochrome P450 reductase (Sirim et al., 2010; Prall et al., 2016). The oxygen-activating domain is positioned above the heme group in the I-helix, which extends the core region of the CYP. This domain contains conserved residues that contribute to the development of a proton groove required for oxygenation *via* breaking the O-O bond and formation of active Fe-O hydroxylating species (Bak et al., 2011; Poulos and Johnson, 2015; Prall et al., 2016). Substrate recognition sites (SRSs) are conserved domains of CYPs located in the globular domain region. The amino acid characteristics of SRSs determine their enzymatic function in terms of substrate specificity, catalytic activity, region-specificity, and shape of the substrate-binding pocket (Gotoh, 1992; Sugimoto and Shiro, 2012).

An enzyme of the CYP716 family is a member of the CYP85 clan of CYPs, which corresponds to non-A type P450s typically considered housekeeping enzymes, providing an evolutionary context for this clade’s origin in the primary metabolism of triterpenoid compounds (Nelson and Werck-Reichhart, 2011; Miettinen et al., 2017). It has been isolated from a wide range of plant families that are functionally characterized as triterpene oxidases. CYP716 is a key enzyme family involved in the biosynthetic pathway for diversifying triterpenoid products in eudicot plants. The CYP716A subfamily is highly conserved in this enzyme family (Fukushima et al., 2011; Fukushima et al., 2013; Miettinen et al., 2017). The modification catalyzed by this subfamily is mainly a three-step oxidation of α-amyrin, β-amyrin, and lupeol at the C-28 position into highly valued compounds such as ursolic acid, oleanolic acid, and betulinic acid, respectively (Carelli et al., 2011; Fukushima et al., 2011; Yasumoto et al., 2016; Miettinen et al., 2017; Yasumoto et al., 2017; Sandeep et al., 2019). For example, CYP716A12 from the model legume *M. truncatula*, CYP716A15 from *Vitis vinifera*, CYP716A48 from *Olea europaea*, and CYP716A49 from *Beta vulgaris* are known enzymes that catalyze this three-step oxidation process. However, there are some CYP716A subfamily enzymes that have been found to catalyze other triterpenoid oxidation reactions, including C-16α and C-22α oxidations (Yasumoto et al., 2016), C-16β of β-amyrin (Tamura et al., 2017), and C-3 oxidations of β-amyrin (Moses et al., 2015). On the other hand, significant differences in catalytic activity and substrate specificity of these enzymes result in heterologous production of different oxidized triterpenoids in transgenic yeast (Suzuki et al., 2018; Dale et al., 2020). Additionally, cytochrome P450s catalytic activity and substrate specificity are considered rate-limiting factors in triterpenoid biosynthesis, affecting the production of C-28 oxidized triterpenoids in heterologous hosts (Zhou et al., 2016; Czarnotta et al., 2017; Suzuki et al., 2018; Jin et al., 2019). Numerous C-28 oxidized triterpenoids have been identified as precursors to various bioactive triterpenoids of commercial interest, including platycodin D, which has been reported to have anti-cancer and anti-viral properties (Liu, 1995; Jeon et al., 2019; Kim et al., 2021). Onjisaponin F is used for increased nasal anti-influenza virus IgA antibody titers (Li et al., 2016; Peng et al., 2020), and QS-21 is used in clinical trials against HIV and malaria as a vaccine adjuvant (Kensil et al., 1998; Liu et al., 2002; Zhu and Tuo, 2016).

Despite their wide distribution and many CYP716A subfamily enzymes being functionally characterized, their crystal structure is not yet available. Little is known about the molecular mechanisms that produce significant differences in the catalytic activity and substrate specificity of these subfamilies (Yasumoto et al., 2016; Miettinen et al., 2017; Suzuki et al., 2018). However, bioinformatics approaches integrated with knowledge from other CYP families may alleviate these problems (Fukushima et al., 2013; Seki et al., 2015; Miettinen et al., 2017). This computerized analysis is less laborious and takes less time to identify potential candidate amino acid residues likely to be involved in catalytic activity of CYP716 family enzyme than the traditional approach such as random mutagenesis.

Our research aimed to identify key amino acid residues for improving the catalytic activity and substrate specificity of CYP716As in *S. cerevisiae* triterpenoid production. We chose CYP716A12, isolated from a model legume, *M. truncatula*, to achieve this goal and to serve as a model for this study. CYP716A12 is the first enzyme in the CYP716A subfamily to be functionally characterized. This enzyme catalyzes regiospecific oxidation at C-28 of α-amyrin, β-amyrin, and lupeol (Fukushima et al., 2011; Suzuki et al., 2018). In this study, the key amino acid residues in the ligand-binding site of CYP716A12 required for the catalytic activity of enzyme and substrate specificity were investigated through homology modeling, structural alignment, *in silico* site-directed mutagenesis, and a docking profile from molecular docking analysis. To elucidate the results of the bioinformatics analysis, *in vivo* functional analyses of candidate mutants were performed. Different product profiles were observed in *S. cerevisiae* engineered to harbor mutant CYP716A12 enzymes when key amino acids at ligand-binding sites were substituted. We also successfully designed CYP716A1 and CYP716A2 to improve the catalytic activity at C-28 against triterpene backbones based on the results of CYP716A12 variants. Our findings may help enhance the expected production of desired triterpenoids by protein engineering.

## Materials and methods

### Chemical authentic standards

All triterpenoid standards (α-amyrin, uvaol, ursolic acid, β-amyrin, erythrodiol, oleanolic acid, lupeol, betulin, and betulinic acid) were purchased from Extrasynthase (France).

### Homology modeling

We selected six enzymes belonging to the CYP716A subfamily functionally characterized as C-28 multifunctional oxidases: CYP716A1, CYP716A2*,* CYP716A12, CYP716A15, CYP716A48, and CYP716A49. The protein sequences of the selected CYP716As are shown in Supplementary Data S1.1. To explore homologous sequences in the protein structure database, a Protein BLAST (BlastP) search with a BLOSUM62 matrix was performed against the experimental structure sequence in the Protein Data Bank (PDB) database (Madden et al., 1996). The structure model with the highest homology with experimental data was chosen as a template. The target protein sequences were aligned with the model sequence, and construction, refinement, and validation of the model were performed using Modeller 9.21 software (Webb and Sali, 2017). The non-water HETATM residues of the model were included in the homology model. Structure optimization was carried out using a multi-domain assembler (MDA). All homology models were visualized using Chimera 1.14 (Pettersen et al., 2004). The quality of homology modeling was evaluated using VERIFY3D, with at least 80% of the amino acids having scored ≥ 0.2 in a 3D/1D profile (Eisenberg et al., 1997).

### Molecular docking analysis

Molecular docking analysis revealed interactions between CYP716As and their putative ligands (α-amyrin, uvaol, ursolic acid, β-amyrin, erythrodiol, oleanolic acid, lupeol, betulin, and betulinic acid) in order to determine the best match interaction between the two molecules. The chemical structures of the putative ligands were obtained from the Cambridge Structural Database (CSD)^1^ and the PubChem database^2^. The scoring function (docking score) was used to predict the binding affinity between the two molecules after docking analysis. The ligand-binding site area for molecular docking analysis was predicted using the I-TASSER modeling pipeline (Yang et al., 2015). Molecular docking analysis was performed using AutoDock Vina 1.1.2 (Trott and Olson, 2010) *via* Chimera.1.14 (Pettersen et al., 2004) with the box center: 50 × −5 × 15 and box dimensions of 30 × 30 × 30 along X, Y, and Z, respectively, in the receptor coordinate system. The lowest docking score, the shortest distance between the oxidation target sites (C-28 of triterpene backbones), and the heme reaction center to the ferrous iron fragment (Fe^2+^) moiety were selected as criteria for the selection of binding poses (Yuki et al., 2012; Geisler et al., 2013). The catalytic site surroundings of the enzyme, including catalytic site capacity, amino acid side chain position and conformation, ligand orientation, and ligand position, were additionally selected as docking evaluation criteria (docking profiles).

### Structural alignment

CYP716A12 homology modeling was used as the reference structure. For the homology modeling, CYP716A1, CYP716A2, CYP716A15, CYP716A48, and CYP716A49 were matched and aligned with the ClustalX algorithm (Thompson et al., 2002). The residue-residue distance cut-off was set at 5 Å. The residue aligned in a column if it was within the cut-off criteria of at least one other. The results of the analysis were visualized using Chimera.1.14 (Pettersen et al., 2004).

### *In silico* site-directed mutagenesis

Candidate residues for site-directed mutagenesis were decided by using the PyMol 2.0.5 software program (Schrodinger LLC, 2015). The native residue of the target residue in the CYP716A homology model was replaced with a representative amino acid based on its properties; non-polar alanine (A) and tyrosine (Y), non-charged polar glutamine (Q), alkaline lysine (K), acidic amino acid aspartic acid (D), and residues in the loop have been replaced with proline (P). The substitution of proline in the loop increases the stability and kinetics of a protein (Matthews et al., 1987). A hydrogen molecule was added and retrained to maintain hydrogen bond in homology model. The structure was exported in PDB format. The structure of the designed protein was optimized and visualized using Chimera.1.14 (Pettersen et al., 2004). Finally, the quality of the structures was assessed using VERIFY3D (Eisenberg et al., 1997).

### Plasmid construction

Bioinformatic predictions were used to experimentally verify the CYP716A12 mutations. The entry clones of CYP716As, pENTR-CYP716A1, pENTR-CYP716A2, pENTR-CYP716A12, pENTR-CYP716A15, pENTR-CYP716A48, and pENTR-CYP716A49 were obtained from previous studies (Seki et al., 2008; Fukushima et al., 2011; Yasumoto et al., 2016). The mutations were introduced to CYP716A12, CYP716A1, and CYP716A2 by site-directed mutagenesis using the PrimeSTAR^®^ Mutagenesis Basal Kit (TaKaRa Bio, Shiga, Japan). The specific primers used for site-directed mutagenesis are shown in (Supplementary Table S1). Yeast expression clones were constructed using the Gateway LR Clonase II Enzyme Mix (Thermo Fisher Scientific) by transferring the coding sequence (CDS) of CYP716A12 into pELC-GW, (Seki et al., 2008) pYES-DEST52 (Thermo Fisher Scientific), and a gateway-compatible version of pESC-HIS (Agilent Technologies) as destination vectors (Seki et al., unpublished). Yeast expression clones of the other CYP716As were constructed using the same approach, including pELC-CYP716As, pYES-DEST52-CYP716As, and pESC-HIS-CYP716As.

### *In vivo* functional analysis in engineering yeast

*S. cerevisiae* INV*Sc*1 (*MATa his3D1 leu2 trp1-289 ura3-52 MATα his3D1 leu2 trp1-289 ura3-52*; Invitrogen) harboring the expression clones for OSC (pYES3-ADH-*OSC1*, pYES3-ADH-*aAS,* or pYES3-ADH-*LUS*) (Fukushima et al., 2011) were further transformed with the constructed expression clone(s) (pELC-CYP716As, pDEST52-CYP716As, and pESC-HIS-CYP716As) using the Frozen-EZ Yeast Transformation II Kit (Zymo Research, Irvine, CA, United States). Transformants were selected on SD selection medium containing 2% glucose, incubated at 30°C for 3 days. Each selected transformant was cultured in 2 ml of the SD selection medium. It was cultured at 30°C overnight at 200 rpm. The overnight culture (500 μl) was transferred into 5 ml of the same medium broth and then cultured at 30°C for 2 days, shaking at 200 rpm. Yeast cells were harvested and resuspended in 5 ml of an appropriate SD medium containing 2% galactose to induce CYP716As expression and cultured at 30°C for 2 days at 200 rpm. Yeast cultures were then stored at −80°C until extraction was performed. All assays were performed in triplicates (three independent assays were obtained from different colonies to confirm the results). *S. cerevisiae* INV*Sc*1 harbors the OSC expression vector (pYES3-ADH-*OSC1*, pYES3-ADH-*aAS,* or pYES3-ADH-LUS), with the empty vectors pELC-GW, pYES-DEST52, and pESC-HIS used as controls.

### Analysis of triterpenoid production in engineering yeast

To quantify triterpenoid production in the yeast culture, 50 μl of internal standard (uvaol or betulin, 100 ppm in methanol) was added to the yeast culture before extraction. Yeast cultures (cells and spent media, 5 ml) were extracted with 5 ml of ethyl acetate (Wako). The mixture was then vortexed and sonicated for 30 min. After centrifugation at 9,000 g for 5 min, the organic phase was transferred to a new tube using a Pasteur pipette. The extracted samples were evaporated using a centrifugal evaporator for 45 min or until they were dry. This procedure was repeated thrice. The remaining powder was resuspended in methanol (1 ml). The obtained samples were transferred into vials using a new Pasteur pipette. The samples were stored at 4°C until the subsequent analysis.

To quantify triterpenoid production in genetically modified yeast, 50 μl of the sample solution (β-amyrin-producing yeast extracts) or 100 μl of the sample solution (α-amyrin or lupeol-producing yeast extracts) was transferred to vial inserts and then to a centrifugal evaporator for 60 min or until dry. Finally, the evaporated pellet was derivatized with 50 μl of *N*-methyl-*N*-(trimethylsilyl) trifluoroacetamide (Sigma-Aldrich) for 30 min at 80°C before Gas Chromatography-Mass Spectrometry (GC-MS) analysis. Fifty microliters of authentic standard solutions (α-amyrin, uvaol, ursolic acid, β-amyrin, erythrodiol, oleanolic acid, lupeol, betulin, and betulinic acid, 10 ppm in methanol) were applied using the same approach described above.

For quantification of triterpenoid production in engineered yeast, GC-MS analysis using a 5977A MSD (Agilent Technologies) coupled with a 7890B GC system (Agilent Technologies) and a DB-1ms (length 30 m, 0.25 mm internal diameter, 0.25 μm film thickness; Agilent Technologies) capillary column was used for metabolite analysis of sample extracts from lupeol-producing yeast extracts. However, a HP-5ms (length 30 m, 0.25 mm internal diameter, 0.25 μm film thickness; Agilent Technologies) capillary column was used for extracting the sample from α-amyrin or β-amyrin-producing yeast extracts. The injection component and the MSD transfer line were set to 250°C. The oven temperature was programmed as follows: 80°C for 1 min, followed by a rise to 300°C at a rate of 20°C min^−1^, and held at 300°C for 14 min. The carrier gas was helium (He) and the flow rate was 1 ml min^−1^. Mass spectra were recorded by scanning the *m/z* range of 50–750. Peaks were identified by comparing the retention times and mass spectra patterns with those of authentic standards (Fukushima et al., 2011; Yasumoto et al., 2016). The relative concentrations of the oxidized triterpenoids in the extracted samples were calculated by comparing the peak area of the analyte with the peak area of the internal standard. To quantify the concentration of triterpenoids in the engineered yeast, an authentic standard curve (α-amyrin, uvaol, ursolic acid, β-amyrin, erythrodiol, oleanolic acid, lupeol, betulin, and betulinic acid) was constructed. The absolute quantification of triterpenoids in yeast was performed by comparing it with the authentic standard curve (Supplementary Methods S2.1, S2.2; Supplementary Figure S1)**.**

### Statistical analysis

The difference in oxidized triterpenoid levels between the yeast strains was determined using one-way analysis of variance (ANOVA), and the significance of the means was determined using Tukey’s test. In this study, *p-*values less than 0.05 (*p* ≤ 0.05) were considered significant. JASP 0.16 for macOS was used for all statistical analyses (JASP Team, University of Amsterdam, Amsterdam, Netherlands).

## Results

### Comparative protein homology modeling of *M. truncatula* CYP716A12

**FIGURE 1:**
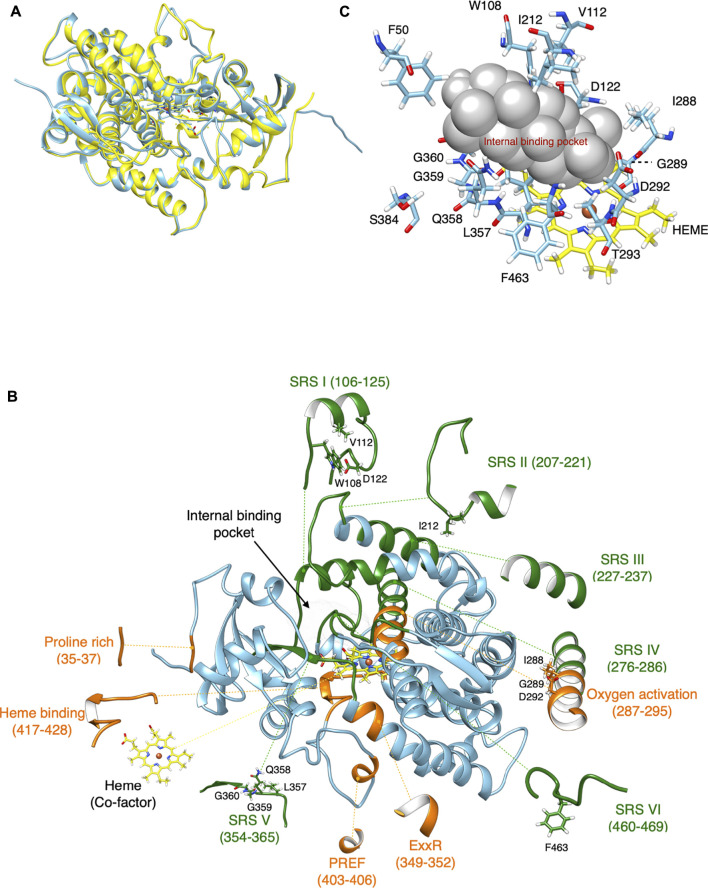
Comparative homology modeling of CYP716A12. **(A)** The retinoic acid bound cyanobacterial CYP120A1(2VE3_A) chain A is the highest model in the similarity of the experimental structure. It was selected as a template model (Yellow) and homology structure of CYP716A12 (blue). **(B)** Homology modeling of CYP716A12 with a predicted ligand-binding site area for molecular docking analysis. Substrate recognition sites (SRS), (green), Signature region (orange). This homology model exceeded the quality criteria verified by VERIFY3D by 89.24%. **(C)** The spatial positions of 15 (15 of 479) candidate amino acid residues were putatively involved in the catalytic activity of the CYP716A12 enzyme in the area of the ligand-binding site *via* a molecular docking analysis and the I-TASSER pipeline.

CYP716A12 was chosen as a model because it is the first CYP716A subfamily enzyme to be functionally characterized; however, no experimental crystal structure for a CYP716A subfamily enzyme is available. Primary protein structure (amino acid sequence) is insufficient for determining enzymatic catalytic activity and substrate specificity. To visualize the location and orientation of each amino acid in protein folding structures, especially at the ligand-binding site, the tertiary structure must be modeled and analyzed (Prall et al., 2016). Consequently, a comparative homology modeling approach was used to construct a homology model of CYP716A12. Homology modeling of CYP716A12 was designed and modeled from its amino acid sequence and an experimental three-dimensional structure of a related homologous protein (template), CYP120A1 (2VE3) chain A (Kuhnel et al., 2008), 38.1% identical (Figure 1A). Unmatched residues in the template were removed during homology modeling.

Finally, 446 amino acid residues were included in the optimized homology model (30–475). In the CYP716A12 homology model, the conserved CYP domain relevant for functional analysis was highlighted. Green highlights indicate substrate recognition sites (SRSI–VI). In contrast, the signature regions are highlighted in orange (Figure 1B). The quality of this homology model exceeded VERIFY3D (Eisenberg et al., 1997) evaluation criteria of 89.24% of residues with an average 3D-1D score greater than 0.2. The same procedure was used to create homology models of CYP716A15 (Supplementary Figure S2A), CYP716A48 (Supplementary Figure S2B), CYP716A49 (Supplementary Figure S2C), CYP716A1 (Supplementary Figure S2D), and CYP716A2 (Supplementary Figure S2E)**.**

### Identification of candidate amino acid residues putatively involved in the catalytic activity of the CYP716A12 enzyme

**FIGURE 2:**
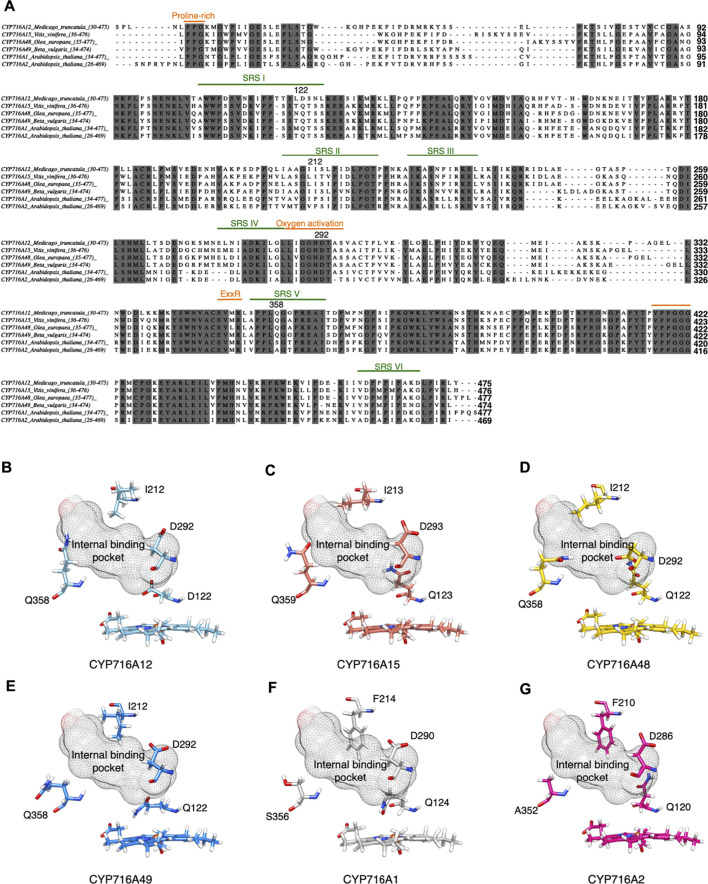
Amino acid residues are predicted to be in the substrate recognition sites and signature region *via* structural alignment of CYP716A12 and selected CYP716As. Structural alignment of selected CYP716As. **(A)** Amino acid sequence alignment of CYP716A12 against selected CYP716As with conserved CYP domains relevant to functional analysis. Predicted substrate recognition sites (SRS), (green); SRSI (106–125); SRSII (207–221); SRSIII (227–237); SRSIV (276–286); SRSV (354–365); SRSVI (460–469), and Signature region (orange); Protein rich (35–37); Oxygen activation (287–295); ExxR (349–352); PREF (403–406); Heme-Binding (417–428) are labeled. Comparison of focusing residues on catalytic activity site of **(B)** CYP716A12 against **(C)** CYP716A15, **(D)** CYP716A48, **(E)** CYP716A49, **(F)**CYP716A1, and **(G)** CYP716A2.

The small spheres of the homology model were placed in the ligand-binding pocket to highlight the substrate-binding region above the heme cofactor using the I-TASSER pipeline (Yang et al., 2015); this area was used for molecular docking analysis (Figures 1B,C). Finally, the spatial positions of 15 candidate amino acid residues (15 of 479) were predicted as ligand binding site residues using I-TASSER pipeline. The majority of these residues were located in the conserved domain at the catalytic site. Among the 15 residues in the conserved CYP domains, 13 were located in SRSI (W108, V112, D122), SRSII (I212), oxygen activation (I288, G289, D292, T293), SRSV (L357, Q358, G359, G360), and SRSVI (F463). Simultaneously, two residues were discovered at the entrance of the binding pocket (F50 and S384) (Figures 1B,C). The catalytic site was not predictive of the amino acids discovered on SRSs III and IV. Further, almost every amino acid residue in these regions is positioned opposite to or away from the ligand-binding site. To investigate the evolutionary and functional relationships that may not be discernible by the comparison of CYP716As, structural alignment using the shape characteristics and structural conformation of the homology model was performed. The amino acid sequence alignment based on structural alignment is shown in Figure 2A. Structural alignment indicated that the amino acid sequences in the signature regions were almost completely conserved. Aspartic acid 292 is a conserved residue in oxygen activation region of the CYP716A subfamily, pointing to the reaction center of the catalytic site (Figures 2B–G). In contrast, the side chains of the remaining conserved residues (I288, G289, T293) pointed away from the ligand-binding pocket.

Interestingly, the amino acid residues in SRS were variable. D122 is an amino acid in SRSI of the CYP716A12 residue; however, glutamine is a conserved residue in several other CYP716As. I212 was detected in SRSII, which is a conserved SRSII residue in CYP716As. Phenylalanine is conserved in this region, except in CYP716A1 and CYP716A2. On CYP716As SRS V, Q358 is a conserved residue. The conserved residues in these aligned CYP716A1 and CYP716A2 are S356 and A352, respectively. F463, located in SRS VI, is not a structurally conserved residue; however, methionine (M) is conserved in CYP716A15 (M464), CYP716A48 (M463), and CYP716A49 (M463).

The most well-characterized CYP716A catalyzes a three-step oxidation reaction at the C-28 position of β-amyrin, α-amyrin, and lupeol, yielding hydroxyl, aldehyde, and carboxylic moieties. Nonetheless, there is a difference between its catalytic activity and substrate specificity. In comparison, some CYP716A enzymes oxidize triterpene backbones at other carbon positions (Fukushima et al., 2011; Miettinen et al., 2017; Yasumoto et al., 2017). As a result, the predicted residues may directly affect catalytic mechanisms and enzymatic activity. Thus, all of the predicted ligand-binding site residues were considered candidates for introducing advantageous amino acid mutations.

### Screening candidate amino acid residues for site-directed mutagenesis

To elucidate the substrate contacts of CYP716A12 that could affect the catalytic activity of the enzyme, molecular docking analysis was performed on the wild-type and mutant versions of the candidate residues identified above. *In silico* site-directed mutagenesis was used to screen for key amino acid residues on ligand-binding sites involved in the catalytic activity and substrate specificity of the enzyme. Then, a docking analysis of molecules was performed. The docking profiles were modified when only four of the fifteen native amino acid residues on the ligand-binding site were replaced.

**FIGURE 3:**
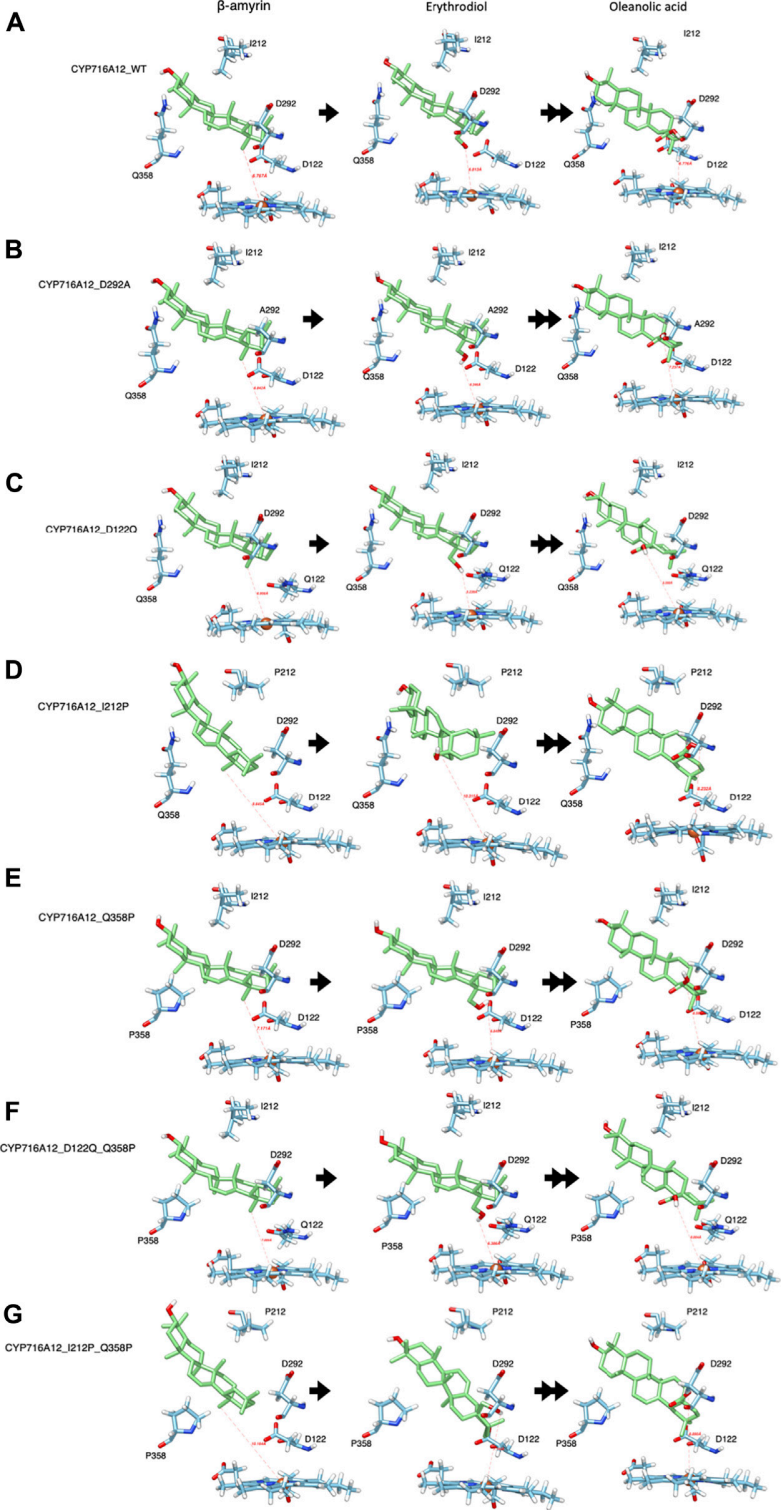
Representative interactions of selected CYP716A12 and its variants against the well-known substrate β-amyrin backbone and its derivatives (Erythrodiol and oleanolic acids). The structure homology modeling showed β-amyrin backbone and its derivatives in wild type of **(A)** CYP716A12, and CYP716A12 variant **(B)** CYP716A12_D292A, **(C)** CYP716A12 _D122Q, **(D)** CYP716A12_I212P, **(E)** CYP716A12_Q358P, **(F)** CYP716A12_D122Q_Q358P, and **(G)** CYP716A12_I212P_Q358P.

When D292 on oxygen activation region was substituted, the docking profile was quite similar to that of the wild-type CYP716A12 (Figure 3A). Nonetheless, when D292 was substituted with alanine, the hydrophobicity of the amino acid residue and environment of the ligand-binding pocket were altered (Figure 3B). Because of its location in the oxygen activation region, this amino acid residue may have an effect on the oxygen activation and electron transfer processes of the enzyme affecting its catalytic activity and substrate specificity.

Similarly, the docking profiles changed when the amino acid residues at SRSI, II, and V were substituted. When D122 was replaced with glutamine, the direction and distance from the target site (C-28) were straighter than those centered on the heme-Fe^2+^ reaction center. The terms distal to the polarity and distance from the ligand-binding pocket were replaced with this substitution (Figure 3C). When I212 was replaced with proline in SRSII, the binding orientation and distance to the substrate changed. Position of C-28 in the reaction center was also altered (Figure 3D). When proline 358 was substituted for Q358 at SRS V, C-28 was oriented more directly toward the reaction center. The capacity of the ligand-binding pocket increased in part because of a change in the direction of the side chain (Figure 3E).

A Q358 (SRSV) was located on the opposite location with D122 (SRS I) and isoleucine 212 (SRSII). As previously stated, when these amino acid residues were replaced, docking profiles were altered. As a result, it is reasonable to speculate that the double residue replacement of the residues on either side of the ligand-binding site is essential for altering the catalytic activity of enzyme and substrate specificity. Thus, we designed a replacement variant of CYP716A12 Q358P with Q122 or P212, resulting in the formation of CYP716A12_D122Q_Q358P and CYP716A12_I212P_Q358P. The substrate orientation profile in the binding pocket of the CYP716A12_D122Q _Q358P variant did not differ significantly from the substituted residue, as previously stated. On the other hand, the properties, environment, and capacity of the ligand-binding site were altered (Figure 3F). CYP716A12_I212P_Q358P has a β-amyrin-binding orientation on the docking profile, similar to that of a single isoleucine 212 substitution; however, the orientation of erythrodiol binding in this variant was significantly altered (Figure 3G).

Interestingly, the comparison of the four substituted residues with the homology model of CYP716A12 revealed a docking profile distinct from that of the other selected CYP716As (Figures 2B–G). The selected CYP716A molecular docking analysis of the β-amyrin backbone was shown in Supplementary Figure S3. In all homology models using the docking profile, the ligand-target sites (β-amyrin and erythrodiol) were within the range and orientation for site-specific oxidation. A homology model revealed a similar docking profile for ligands in wild-type CYP716A12 (Supplementary Figure S3A) and CYP716A15 (Supplementary Figure S3B), whereas it was different from CYP716A48 (Supplementary Figure S3C) and CYP716A49 (Supplementary Figure S3D), respectively.

The same analyses were performed on molecular docking analysis to reveal the docking profile against the other putative substrates, α-amyrin (Supplementary Figures S4, S5), and lupeol backbones (Supplementary Figures S6, S7)**.** This analysis revealed differences in the docking profile of the engineered variant of CYP716A12, which further altered the catalytic activity of enzyme and substrate specificity.

The preliminary results obtained from homology modeling, structural alignment, *in silico* site-directed mutagenesis, and the docking profile from molecular docking analysis suggested a strategy for gaining a better understanding of the amino acid differences at each CYP716A ligand-binding site. Properties of the ligand-binding site were altered when candidate key amino acid residues were replaced. This would also affect the ability of the enzyme to generate enormous variation in its catalytic activity and substrate specificity. Altering the properties of important residues and the environment surrounding the ligand-binding site may significantly affect substrate recognition, and binding may affect the catalytic activity (Narayan et al., 2015; Blaha-Nelson et al., 2017; Li et al., 2017; Baek et al., 2020; Sun et al., 2020). We hypothesized that altering the docking profile would affect substrate recognition, thereby affecting the binding pocket and triterpene backbone interaction and, in turn, the catalytic activity of enzyme.

To test our hypothesis, we performed an *in vivo* functional analysis to determine the catalytic activity of enzyme and substrate specificity to provide a more detailed explanation of the enzymatic activity and quantification of the desired product.

### *In vivo* functional analysis in engineered yeast harboring the CYP716A sub-family enzymes

**FIGURE 4:**
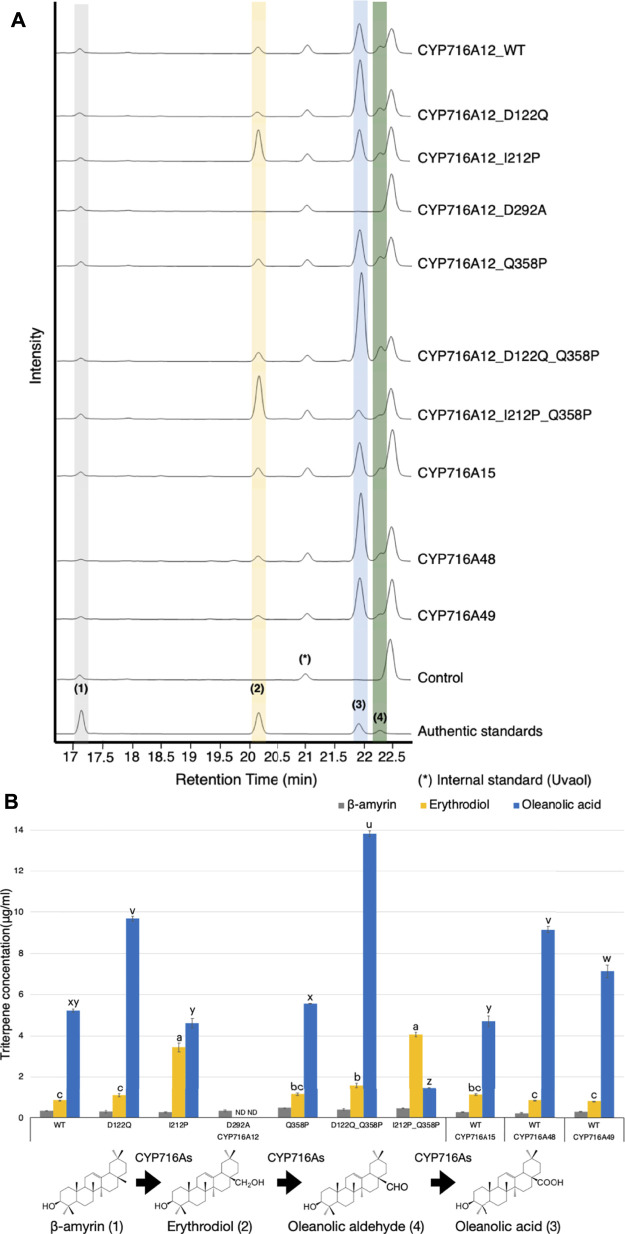
*In vivo* activities of selected CYP716As and CYP716A12 and its variants against triterpene skeletons. **(A)** Total ion chromatograms (TICs) of extracts from yeast harboring one *bAS* expression vector for producing β-amyrin and three CYPs expression vectors (pELC-CYP716As, pYES-DEST52-CYP716As, and pESC-HIS-CYP716As) were used. **(B)** The quantification of triterpene concentration in yeast harboring CYP716As against β-amyrin skeletons. Quantitation and error bars correspond to the mean and standard deviation, respectively. Data are representative of at least three biological replicates (*n* = 3). Letters indicate statistical differences between the different oxidized triterpenoids levels (a–c: erythrodiol levels, u–z: oleanolic acid levels) for each sample (one-way ANOVA; Tukey’s post-hoc test, *p* ≤ 0.05). ND, not detected.

When candidate key amino acid residues at the ligand-binding site were substituted, integration results using bioinformatics approaches altered the pattern of the docking profile. An *in vivo* functional analysis of the enzyme in yeast was performed to substantiate the bioinformatics prediction and study their enzymatic activities. Yeast strains expressing one β-amyrin synthase (*bAS*) expression vector together with each of the three CYP expression vectors of CYP716A12 wild-type and their variants (D292A, D122Q, I212P, Q358P, D122Q_Q358P, or I212P_Q358P), selected CYP716As (CYP716A15 wild-type, CYP716A48 wild-type, and CYP716A49 wild-type), or the control vector (empty expression vector) were generated. To observe the triterpenoid profile of β-amyrin-producing yeast extract, product analysis was carried out using GC-MS (Figure 4A). The same approach was applied to yeast, which produces the other triterpene backbones, α-amyrin (Supplementary Figure S8A), and lupeol (Supplementary Figure S10A), harboring three target CYP expression vectors. We observed that when native amino acids at SRSs were substituted, the metabolite profile of CYP716A12 variants against all triterpene backbones was altered in yeast (Figure 4A; Supplementary Figures S8A, S10A).

The concentration of triterpenes in a yeast culture harboring CYP716As against β-amyrin backbones was quantified to elucidate the *in vivo* catalytic activity and substrate specificity of the engineered yeast by comparing the production of triterpenoids. As a result, the concentration of the C-28 oxidized triterpenoid product (oleanolic acid) was significantly higher in the yeast culture harboring wild-type CYP716A12 (5.22 ± 0.08 μg/ml) than in the yeast culture harboring CYP716A15 (4.70 ± 0.25 μg/ml). However, the production of oleanolic acid in the yeast culture harboring CYP716A48 (9.14 ± 0.15 μg/ml) and CYP716A49 (7.15 ± 0.31 μg/ml) were higher than in the yeast culture harboring CYP716A12 wild-type (Figure 4B). In comparison, the triterpene concentrations of CYP716A12 variants were significantly different from those of the wild type. The production of oleanolic acid was increased when CYP716A12 variants D122Q (1.85 times), Q358P (1.06 times), or D122Q_Q358P (2.65 times) were co-expressed in engineering yeast compared with wild-type (Figure 4B). The highest quantities of oleanolic acid were found in the CYP716A12 variant, D122Q_Q358P, at 13.82 ± 0.13 μg/ml. Simultaneously, when the CYP716A12 variants, I212P or I212P_Q358P were co-expressed in yeast, erythrodiol production was significantly increased (Figure 4B).

When CYP716A12 variants were expressed in α-amyrin-producing yeast, the concentration of the C-28 oxidized triterpenoid product (ursolic acid) in the yeast culture harboring wild-type CYP716A12 (2.58 ± 0.17 μg/ml) was lower than that of other selected CYP716As. The highest concentration of ursolic acid (8.57 ± 0.23 μg/ml) was found in yeast harboring the wild-type CYP716A48 (Supplementary Figure S8B). The concentration of ursolic acid increased in the yeast strain harboring variants of CYP716A12, D122Q, and D122Q_Q358P (Supplementary Figure S8B). Although this yeast system produces α-amyrin as a major product, it could be detected as a minor product (Suzuki et al., 2018), thus determining the substrate specificity of CYP716As in yeast. This occurs since there is no α-amyrin synthase that produces only α-amyrin. In a yeast co-expressing α-amyrin synthase and CYP716As, we could detect both derivatives [from α-amyrin (Ursane-type), and β-amyrin (Oleanane-Type)], being α-amyrin was more abundant. As a result of this finding, the yeast strains harboring the wild types CYP716A15, CYP716A48, and CYP716A49 were preferred for producing ursolic acid (Ursane-type) over oleanolic acid (Oleanane-type). Surprisingly, yeast strains harboring CYP716A12 or its variants have been found to preferentially produce oleanolic acid (Oleanane-type) over ursolic acid (Ursane-type). These results indicate that while key residues in CYP716A12 were replaced, substrate specificity remained unchanged; however, the catalytic activity of the enzyme was improved when key amino acid residues were substituted (Supplementary Figures S8A,B, S9).

Although the engineered yeast produced lupeol, the concentration of the C-28 oxidized triterpenoid product (betulinic acid) was lower (1.39 ± 0.10 μg/ml) in the yeast culture harboring wild-type CYP716A12 than in the other selected CYP716As. However, the betulinic acid concentration was enhanced in yeast containing the CYP716A12 variants D122Q, Q358P, and D122Q_Q358P. The highest concentration of betulinic acid (8.75 ± 0.17 μg/ml) was found in the CYP716A12 variant D122Q_Q358P (Supplementary Figures S10A,B)**.**

Surprisingly, when the amino acid in the residual oxygen activation region 292 (D292A) was replaced, no oxidized triterpenoid products were detected in any yeast strain producing triterpene backbones (β-amyrin, α-amyrin, and lupeol) harboring this variant (Figure 4; Supplementary Figures S8–S10).

Taken together, these data support our hypothesis that the observed changes in product profiles were the result of a change in the docking profile after performing *in silico* site-directed mutagenesis and molecular docking analysis.

### Improving the C-28 catalytic activity of *A. thaliana* CYP716A1 and CYP716A2 inspired by CYP716A12 variants

**FIGURE 5:**
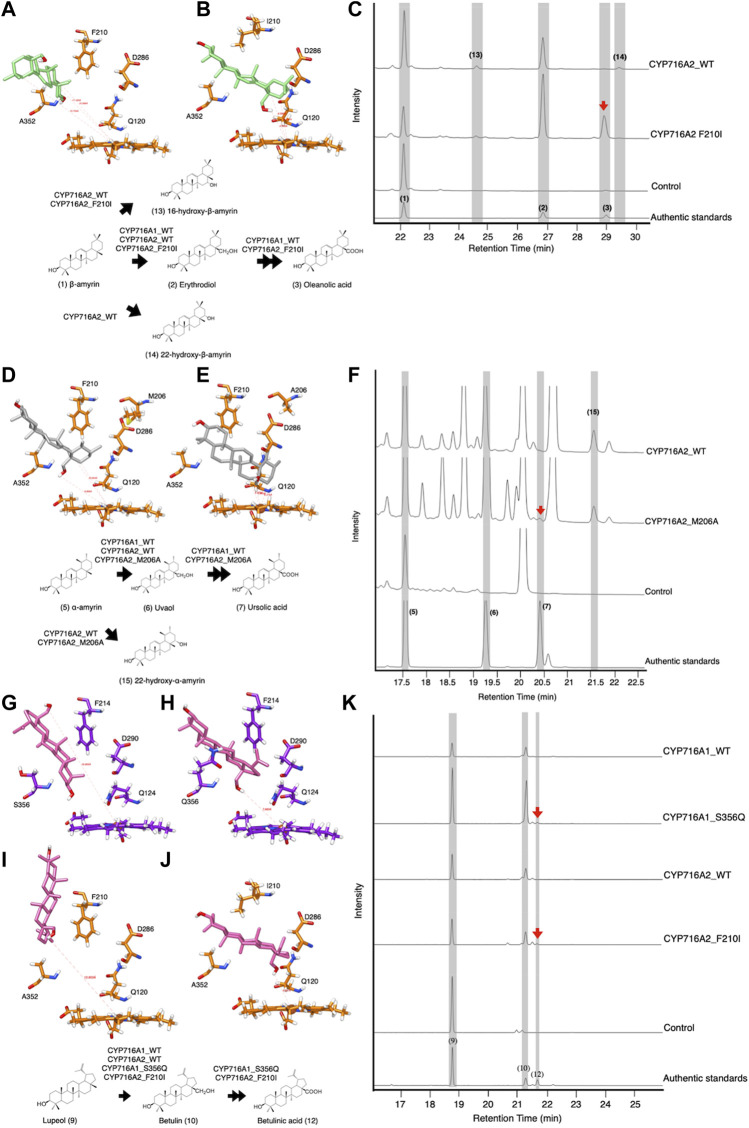
*In-silico* study and *In vivo* activity of CYP716A1 and CYP716A2 toward triterpene skeletons in co-expressing yeast strain. Representative interactions of **(A)** CYP716A2 wild-type, and **(B)** it variant (CYP716A2_F210I) against erythrodiol backbone. **(C)** TICs of extracts from yeast harboring one *bAS* expression vector for producing β-amyrin and three CYPs expression vectors of CYP716A2 or its variant (CYP716A2_F210I). Representative interactions of **(D)** CYP716A2 wild-type, and **(E)** it variant (CYP716A2_M206A) against uvaol backbone. **(F)** TICs of extracts from yeast harboring aAS expression vector for producing α-amyrin as a major substrate and three CYPs expression vectors CYP716A2 or its variant (CYP716A2_M206A). Representative interactions of **(G)** CYP716A2 wild-type, and **(H)** it variant (CYP716A2_M206A) **(I)** CYP716A1 wild-type, and **(J)** it variant (CYP716A1_S356Q) against betulin backbone. **(K)** EIC of extracts from yeast harboring one *LUS* expression vector for producing lupeol and three CYPs expression vectors of CYP716A2 or CYP716A2_M206A or CYP716A1 or CYP716A1_S356Q. Data are representative of at least three biological replicates (*n* = 3).

A previous study demonstrated that CYP716A1 and CYP716A2 catalyzed the C-28 oxidant against a similar triterpene backbone in several enzymes belonging to the CYP716A subfamily (Yasumoto et al., 2016). In engineered yeast harboring CYP716A1, oleanolic acid and ursolic acid are detected when co-expressed with β-amyrin synthase and α-amyrin synthase, respectively. However, these products were undetectable in the engineered yeast expressing CYP716A2. Surprisingly, a variety of alcohols, including erythrodiol, 16-hydroxy-β-amyrin, 22α-hydroxy-β-amyrin, and 22α-hydroxy-α-amyrin, are detected in engineered yeast harboring CYP716A2 (Yasumoto et al., 2016). Nevertheless, betulinic acid is not detectable in lupeol-producing yeast harboring CYP716A1 or CYP716A2 (Yasumoto et al., 2016). When the amino acid in the SRS of CYP716A12 was replaced, the product profile in C-28 oxidases was altered depending on the triterpene backbone. Specifically, variants with amino acids in SRSII and SRSV were substituted. The amino acid sequences on SRS II and SRS V of CYP716A1 and CYP716A2 were considered to be structurally aligned with CYP716A12 to increase the catalytic activity at C-28 of triterpene backbones (Figure 2A). When isoleucine was substituted for F210 in SRS II of CYP716A2, the docking profile of the variant against erythrodiol was altered. The C-28 direction and distance were almost perfectly centered at the reaction center (Figures 5A,B). *In vivo* functional analysis of β-amyrin-producing yeast harboring this variant revealed the production of oleanolic acid and 16-hydroxy-β-amyrin. In comparison, 22-hydroxy-β-amyrin was not detected (Figure 5C).

Additionally, when alanine (A) was substituted for M206 on SRS II of CYP716A2 and the docking profile was compared to that of the wild-type, the docking profile of the variant was altered (Figures 5D,E). Ursolic acid and 22α-hydroxy-α-amyrin production were detected in α-amyrin-producing yeast harboring this variant (Figure 5F). Likewise, when glutamine was substituted for S356 on CYP716A1 SRSV, the docking profile against betulin was altered (Figures 5G,H). Similarly, the docking profile indicated that the orientation of betulin in the ligand-binding pocket of CYP716A2 was different in the variant in which isoleucine was substituted with F210 on SRS II. The direction and distance of C-28 were determined using points centered on the Fe^2+^ moiety heme reaction center (Figures 5I,J). However, betulinic acid production was observed in the engineered lupeol-producing yeast harboring these variants (CYP716A1_S356Q and CYP716A2_F210I) (Figure 5K).

## Discussion

The CYP716 enzyme family has been identified in a diverse array of plant species. These family members catalyze a variety of modifications of the triterpene backbone. The three-step site-specific oxidation at the C-28 position of the triterpene backbone is the most common modification of CYP716A found in triterpenoids (Carelli et al., 2011; Fukushima et al., 2011; Yasumoto et al., 2016; Miettinen et al., 2017; Yasumoto et al., 2017; Sandeep et al., 2019). Our analysis demonstrates that while the structural alignment of the signature region is highly conserved between CYP716As, their SRS is highly variable. Variations in SRS dictate the diversity of enzyme functions in terms of substrate specificity, catalytic activity, regio-specificity, and substrate-binding pocket characteristics (Gotoh, 1992; Sugimoto and Shiro, 2012).

We have identified the critical amino acid responsible for the significant difference between catalytic activity and substrate specificity in C-28 oxidases, which has remained unknown in previous studies. By integrating bioinformatics techniques and *in vivo* functional analysis in yeast, we were able to identify critical amino acids responsible for the disparity between the catalytic activity and substrate specificity in C-28 oxidases. We found that changing the properties of a key amino acid residue in the ligand-binding site could alter the product profile of CYP716A12, in particular, the key amino acids found on SRSs.

Aspartic acid 122, located in SRSI of CYP716A12, is a key residue for increasing the catalytic activity of the enzyme. This residue can be modified to control the direction of the target site toward the reaction center, particularly in the β-amyrin and lupeol backbones, thereby increasing the accumulation of oxidized products. Notably, in engineered yeast, there was a significant increase in the production of oleanolic acid and betulinic acid.

Isoleucine 212 and nearest-neighbor residues in SRSII of CYP716A12 significantly affected the C-28 oxidation efficiency of the triterpene backbones. The amino acid side chain of a residue in this region may alter the binding orientation of the triterpene backbone in the internal binding pocket, thereby altering the yield of the triterpene-oxidized products in yeast.

Although modifying glutamine 358 in SRSV does not affect the orientation of the substrate, it can slightly alter the catalytic activity of an enzyme. Specifically, the CYP716A12_D122Q_Q358P variant enhanced the catalytic activity of the enzyme and expanded its ligand-binding pocket capacity. The observed increase in the oxidized triterpenoid yield was due to the double substitution of two critical residues. With our current data, we can speculate that the Q358P variant of CYP716A48 will be more active than its wild type, still this needs to be confirmed by experimental work. In α-amyrin-producing yeast, the Q358P and D122Q_Q358P variants accumulate more alcohol intermediate (Uvaol) than the acid product (Ursolic acid). Molecular docking analysis suggests that the capacity and environment of the ligand-binding site were changed when the residues Q358P were substituted. It would help to improve the binding efficiency of α-amyrin (substrate) at the ligand-binding site. Therefore, uvaol (oxidized product) was detected at a high level in both co-expression with two variants. To design the enzyme with the function that can be modified to stop at the alcohol (Uvaol) (C-28 hydroxy function), molecular docking analysis suggests that substituting I212 by proline might help to improve alcohol production because it might be interrupt the binding efficiency of the substrate. Therefore, triple substitution (D122Q_I212P_Q358P) might also help to stop the production at alcohol (Uvaol).

Surprisingly, the CYP716A12_I212P_Q358P variant increased the ligand-binding site capacity. However, the amino acid side chain at residue 212 (proline 212) impaired the binding efficiency of the substrate. Therefore, the catalytic activity of this enzyme might be limited.

Aspartic acid 292 is a critical amino acid residue in the oxygen activation domain. The orientation of the substrate, as determined by the docking profile, indicated that this possibility remained unchanged when alanine was substituted for this residue. However, the amino acid properties of the ligand-binding pocket were altered, which would obstruct the contraction of the enzyme with the substrate. Additionally, binding of the substrate to the enzyme-binding pocket was also inhibited. No oxidation product was detected in the yeast producing triterpene backbones harboring this variant during *in vivo* functional analysis. As a result, this residue was designated as critical for enzyme activity. This position in relation to aspartic acid residue 301, located in the oxygen activation region, was critical in determining substrate specificity and CYP2D6 activity in a previous study (Ellis et al., 1995).

Inspired by the engineering achievements of CYP716A12, we demonstrated that modifying the properties of SRS II residues (M206A, F210I) can increase the catalytic activity of CYP716A2 for heterologous production of oxidized C-28 triterpenoids in yeast. Nonetheless, it impaired the CYP716A2 variant site-specific oxidation activity at C-22 of the β-amyrin backbone. Similarly, engineering SRS V serine 356 caused CYP716A1 to alter the catalytic activity on the lupeol backbone. To modify CYP716A1 and CYP716A2 more functionally efficient at C-28 oxidases, we suggest engineering by double mutation at SRS II and SRS V.

Our results suggest a strategy for increasing knowledge about critical amino acid residues involved in the catalytic activity and substrate specificity of CYP716A subfamily enzymes to increase triterpenoid production in yeast. However, the specific catalytic mechanism of the enzyme following substitution of critical residues is currently unknown.

In summary, our work identified critical amino acid residues in the CYP716A subfamily that contribute to its catalytic activity and substrate specificity. This study provides a framework for enhancing heterologous production of oxidized C-28 triterpenoids in yeast through enzyme engineering. We demonstrated the enormous utility of this approach by engineering *A. thaliana* CYP716A1 and CYP716A2 to modify the catalytic activity at C-28 on triterpene backbones. Altering the obtained results has numerous applications in protein engineering towards the designed production of triterpenoids making it is suitable for various biotechnological and pharmaceutical applications.

## Data Availability

The original contributions presented in the study are included in the article/Supplementary Materials, further inquiries can be directed to the corresponding authors.

## Footnotes

1
https://www.ccdc.cam.ac.uk/theccdcprofile/.

2
https://pubchem.ncbi.nlm.nih.gov.

## Abbreviations

SRSs, Substrate Recognition Sites; OSC, Oxidosqualene cyclase; CYPs, Cytochrome P450 monooxygenases; CYP716As, selected CYP 716 subfamily A; TICs, Total ion chromatograms; EIC, Extracted ion chromatogram; GC-MS, Gas Chromatography-Mass Spectrometry; bAS, β-amyrin synthase; aAS, α-amyrin synthase; LUS, lupeol synthase.
