# Supplementary material for: Identification of key amino acid residues toward improving the catalytic activity and substrate specificity of plant-derived cytochrome P450 monooxygenases CYP716A subfamily enzyme for triterpenoid production in Saccharomyces cerevisiae
Source: Front Bioeng Biotechnol. 2022 Aug 19;10:955650. doi: 10.3389/fbioe.2022.955650 (PMC9437279; doi:10.3389/fbioe.2022.955650)
Supplement: Supplementary file 1 [file DataSheet1.pdf]

Supplementary Material

Supplementary Contents

1. **Supplementary data**

   1.1 The protein sequence of the CYP716A subfamily used in this study

   1.2 The specific primers for site-directed mutagenesis

   1.3 List of variants

2. **Supplementary methods**

   2.1 GC–MS analysis for the functional analysis of CYP716A2 in yeasts producing β-amyrin

   2.2 Standard curve for calculating the quantifications of triterpene concentrations in engineered yeast

3. **List of tables**

   Table S1. Specific primer for site-directed mutagenesis

   Table S2. CYP716As wild-type, Variants, and mutations in *in vivo* functional analysis

4. **List of Figures**

   Supplementary Figure 1 The authentic standard curve of the triterpenoid.

   Supplementary Figure 2 Comparative homology modeling of CYP716As with identified internal binding pocket.

   Supplementary Figure 3 Representative interactions of selected CYP716As against β-amyrin backbone and its derivatives (erythrodiol and oleanolic acids).

   Supplementary Figure 4 Representative interactions of CYP716As against α-amyrin backbone and its derivatives (uvaol, ursolic acid).

   Supplementary Figure 5 Representative interactions of CYP716A12 and its variants against α-amyrin backbone and its derivatives (uvaol, ursolic acid).

   Supplementary Figure 6 Representative interactions of selected CYP716As against lupeol backbone and its derivatives (betulin, betulinic acid).

   Supplementary Figure 7 Representative interactions of CYP716A12 and its variants against lupeol backbone and its derivatives (betulin, betulinic acid).

Supplementary Figure 8 *In vivo* activities of selected CYP716As and CYP716A12, and its variants against α-amyrin skeletons.

Supplementary Figure 9 Comparison of triterpene concentration from extracts of yeast producing α-amyrin (Ursane-type) as a major substrate and β-amyrin (Oleanane-Type) as a minor substrate.

Supplementary Figure 10 *In vivo* activities of selected CYP716As and CYP716A12, and its variants against lupeol skeletons.

1 Supplementary data

1.1 The protein sequence of the CYP716A subfamily used in this study

> A_AtCYP716A1_*Arabidopsis_thaliana* (accession no. NM_123002.2)
MYMAIMIILFLSSILLSLLLLLRKHLSHFSYPNLPPGNTGLPLIGESFSFLSAGRQGHPEKFI
TDRVRRFSSSSSCVFKTHLFGSPTAVVTGASGNKFLFTNENKLVVSWWPDSVNKIFPSSM
QTSSKEEARKLRMLLSQFMKPEALRRYVGVMDEIAQRHFETEWANQDQVIVFPLTKKF
TFSIACRSFLSMEDPARVRQLEEQFNTVAVGIFSIPIDLPGTRFNRAIKASRLLRKEVSAIV
RQRKEELKAGKALEEHDILSHMLMNIGETKDEDLADKIIGLLIGGHDTASIVCTFVVNYL
AEFPHVYQRVLQEQKEILKEKKEKEGLRWEDIEKMRYSWNVACEVMRIVPPLSGTFRE
AIDHFSFKGFYIPKGWKLYWSATATHMNPDYFPEPERFEPNRFEGSGPKPYTYVPFGGGP
RMCPGKEYARLEILIFMHNLVNRFKWEKVFPNENKIVVDPLPIPDKGLPIRIFPQSX

> A_AtCYP716A2_*Arabidopsis_thaliana* (accession no. LC106013.1)
MYLTIIFLFISSIIFPLLFFLGKHLSNFRYPNLPPGKIGFPLIGETLSFLSAGRQGHPEKFVTD
RVRHFSSGIFKTHLFGSPFAVVTGASGNKFLFTNENKLVISWWPDSVNKIFPSSTQTSSKE
EAIKTRMLLMPSMKPEALRRYVGVMDEIAQKHFETEWANQDQLIVFPLTKKFTFSIACR
LFLSMDDLERVRKLEEPFTTVMTGVFSIPIDLPGTRFNRAIKASRLLSKEVSTIIRQRKEEL
KAGKVSVEQDILSHMLMNIGETKDEDLADKIIALLIGGHDTTSIVCTFVVNYLAEFPHIY
QRVLEEQKEILNNKDVNEKLTWEDIEKMRYSWNVACEVMRIVPPLAGTFREAIDHFSFK
GFYIPKGWKLYWSATATHKNPEYFPEPEKFEPSRFEGSGPKPYTYVPFGGGSRICPGREY
ARLEILIFMHNLVKRFKWEKVFPKENKLVADPAPIPAKGLPIRIFPQS

> A_MtCYP716A12_*Medicago_truncatula* (accession no. DQ335781.1)
MEPNFYLSLLLLFVSFISLSLFFIFYKQKSPLNLPPGKMGYPIIGESLEFLSTGWKGHPEKFI
FDRMRKYSSELFKTSIVGESTVVCCGAASNKFLFSNENKLVTAWWPDSVNKIFPTTSLDS
NLKEESIKMRKLLPQFFKPEALQRYVGVMDVIAQRHFVTHWDNKNEITVYPLAKRYTFL
LACRLFMSVEDENHVAKFSDPFQLIAAGIISLPIDLPGTPFNKAIKASNFIRKELIKIIKQRRI
DLAEGTASPTQDILSHMLLTSDENGKSMNELNIADKILGLLIGGHDTASVACTFLVKYLG
ELPHIYDKVYQEQMEIAKSKPAGELLNWDDLKKMKYSWNVACEVMRLSPPLQGGFRE
AITDFMFNGFSIPKGWKLYWSANSTHKNAECFPMPEKFDPTRFEGNGPAPYTFVPFGGG
PRMCPGKEYARLEILVFMHNLVKRFKWEKVIPDEKIIVDPFPIPAKDLPIRLYPHKA

> A_VvCYP716A15_*Vitis_vinifera* (accession no. AB619802.1)
MEVFFLSLLLIFVLSVSIGLHLLFYKHRSHFTGPNLPPGKIGWPMVGESLEFLSTGWKGHP
EKFIFDRISKYSSEVFKTSLLGEPAAVFAGAAGNKFLFSNENKLVHAWWPSSVDKVFPSS
TQTSSKEEAKKMRKLLPQFFKPEALQRYIGIMDHIAQRHFADSWDNRDEVIVFPLAKRFT
FWLACRLFMSIEDPAHVAKFEKPFHVLASGLITVPIDLPGTPFHRAIKASNFIRKELRAIIK
QRKIDLAEGKASQNQDILSHMLLATDEDGCHMNEMEIADKILGLLIGGHDTASAAITFLI
KYMAELPHIYEKVYEEQMEIANSKAPGELLNWDDVQNMRYSWNVACEVMRLAPPLQG
AFREAITDFVFNGFSIPKGWKLYWSANSTHKSPECFPQPENFDPTRFEGNGPAPYTFVPF
GGGPRMCPGKEYARLEILVFMHNVVKRFKWDKLLPDEKIIVDPMPMPAKGLPVRLHPH
KP

>A_OeCYP716A48_*Olea_europaea* (accession no. G0244188.1)
MEFFYVSLLCLFVFLISLSLHFLFYKNKSSFSGQIPPGKTGWPVIGESLEFLSNGWKGHPE
KFIFDRIAKYSSYVFRTHLFGEPAAVFCGANGNKFLFSNENKLVQAWWPASVDKVFPSS
NQTSSKEEAVKMRKMLPTFFKPEALQRYVGIMDHIAQRHFSDGWDNKNEVVVFPLAKR
YTFWLACRLFVSVEDPAHVAKFADPFNELASGLISIPIDLPGTPFHRAIKSSNFIRKELVSII
KQRKIDLAEGKASPTQDILSHMLLTSDESGKFMHELDIADKILGLLVGGHDTASSACTFV
VKYLAELPEIYEGVYQEQMEIAKSKAPGELLNWDDIQKMKYSWNVACEVLRLAPPLQG
AFREAITDFMFNGFSIPKGWKLYWSANSTHRNSEFFPEPLKFDPSRFEGSGPAPYTFVPFG
GGPRMCPGKEYARLEILVFMHHLVKRFKWEKLIPDEKIVVDPMPIPAKGLPIRLYPLNA

>A_BvCYP716A49_*Beta_vulgaris* (accession no. FG44256.1)
MELFFLCGLILFLSLSLASLYLLYNHNSTKGYRVPPGTMGWPVVGESLEFLSTGWKGYP
EKFIFDRLSKYAPNQIFKTSILGEKVAVICGAAGNKFLYSNENKLVQAWWPSSVDKIFPS
STQTSSKEESKKMRKLLPNFLKPEALQRYIPIMDTIAIRHMESGWDGKDKVEVFPLAKRY
TFWLACRLFLSIEDPDHVAKFAEPFNDIAAGIISLPVNLPGTPFNRGIKSSNVVRKELRAII
KQRKLDLADGKASTTQDILSHMLLTADEDGRFMTEMDIADKILGLLIGGHDTASAACTF
VVKYLAELPHVYEAVCKEQMEIAKSKAEGELLNWEDIQKMKYSWNVACEVMRLAPPL
QGGFREAISDFMYGGFQVPKGWKLYWSANSTHRNPECFPEPEKFDPSRFEGKGPAPYTY
VPFGGGPRMCPGKEYARLEILVFMHNVVKRFKWEKVLPNEKVIVNPMPIPENGLPVRLF
PHPQIVAA

1.2 The specific primers for site-directed mutagenesis

Table S1. Specific primer for site-directed mutagenesis

Primer name	Forward (5'->3')	Reverse (5'->3')
**CYP716A12_D122Q**	TCTCTTCAATCTAACTTGAAGGAAGAA	GTTAGATTGAAGAGAAGTAGTAGGGAA
**CYP716A12_I212P**	GGAATCCCATCTCTACCAATTGATTTG	TAGAGATGGGATTCCGGCTGCAATTAA
**CYP716A12_D292A**	GGACATGCTACTGCTAGCGTCGCATGC	AGCAGTAGCATGTCCTCCGATCAAAAG
**CYP716A12_Q358P**	CCACTCCCAGGAGGTTTCAGGGAAGCC	ACCTCCTGGGAGTGGAGGGGAAAGTCT
**CYP716A1_S356Q**	CCTCTTCAAGGCACTTTTCGTGAGGCC	AGTGCCTTGAAGAGGAGGAACAATTCT
**CYP716A2_M206A**	ACGGTTGCTACGGGTGTCTTCTCAATC	ACCCGTAGCAACCGTAGTGAATGGCTC
**CYP716A2_F210I**	GGTGTCATTTCAATCCCAATAGATTTA	GATTGAAATGACACCCGTCATAACCGT

2 Supplementary methods

2.1 GC–MS analysis for the functional analysis of CYP716A2 in yeasts producing β-amyrin

Gas chromatography-mass spectrometry (GC-MS) analysis was performed using an HP-5MS capillary column. The injection component and the MSD transfer line were set to 250 °C. The oven temperature was programmed as follows: 150°C for 1 min, followed by an increase to 260°C at a rate of 30°C min^{-1}, followed by an increase to 300°C at a rate of 1°C min^{-1}.

2.2 Standard curve for calculating the quantifications of triterpene concentrations in engineered yeast

Standard curves were used to determine the relationship between the quantities of the two compounds. They are used to calculate the value of an unknown amount (triterpene concentration) of metabolites extracted from engineered yeast with respect to the one that is more easily measured (triterpene standard), which has known concentration. To quantify triterpene concentrations extracted from engineered yeast, an authentic standard curve of triterpene compounds was constructed and the relative quantification of concentration was calculated by comparing the peak area in the authentic standard with the peak area of an internal standard. An authentic standard curve was used to plot the relative peak area of the internal standard versus a varying but known triterpene concentration.

The actual concentration of triterpene in yeast was calculated using the standard curve equation.

3 Supplementary Figures

3.1 The authentic standard curve of the triterpenoid

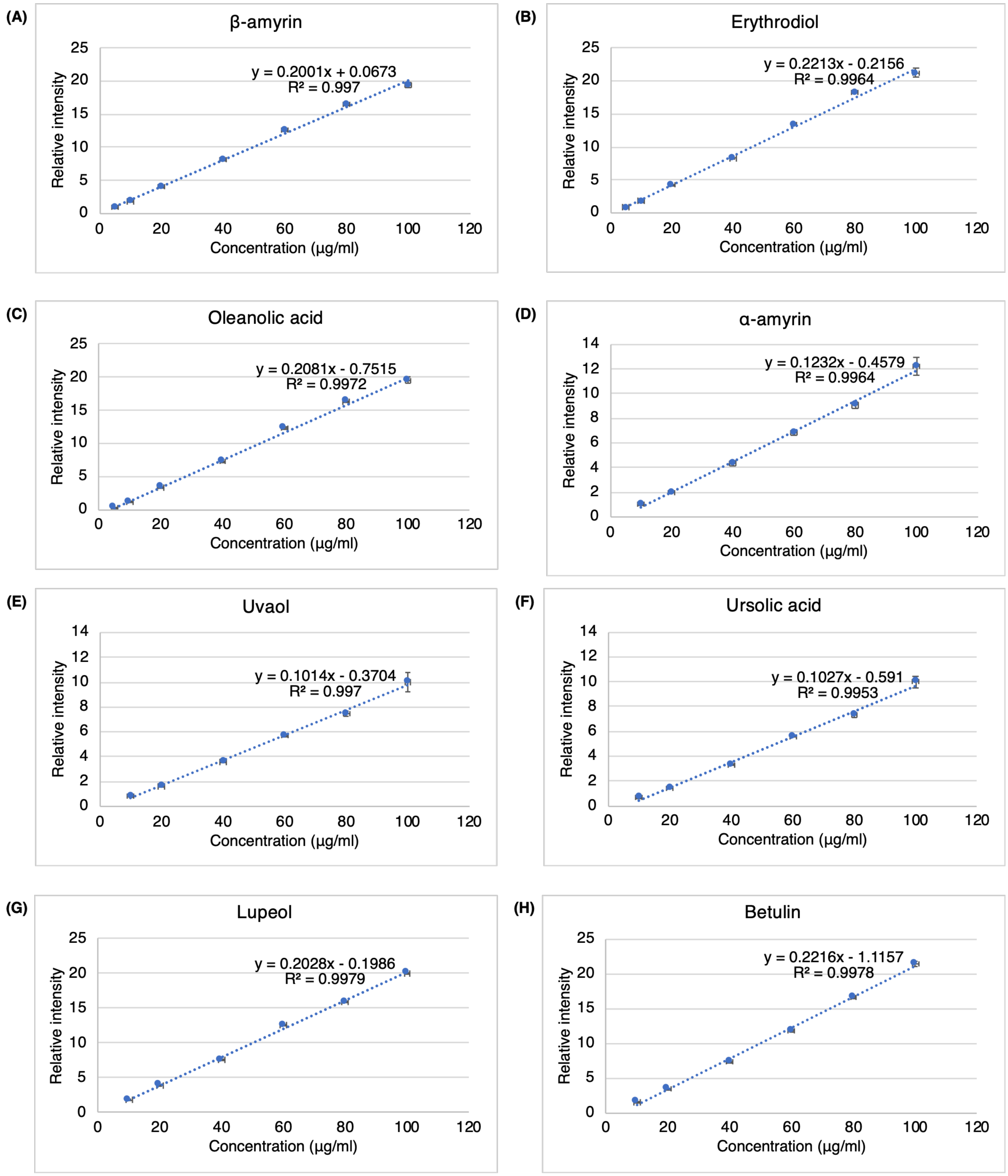

(I)

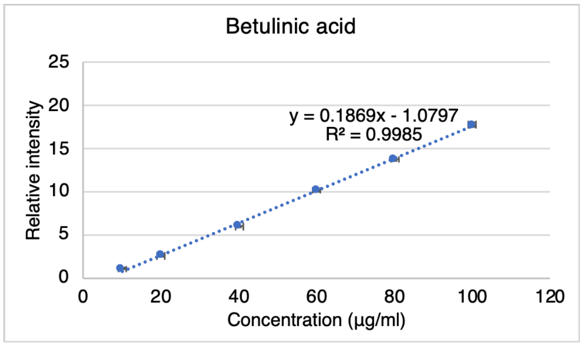

Supplementary Figure 1 The authentic standard curve of the triterpenoid.

(A) β-amyrin, (B) erythrodiol, (C) oleanolic acid, (D) α-amyrin, (E) uvaol, (F) ursolic acid, (G) lupeol, (H) betulin, and (I) betulinic acid. Data are representative of at least three biological replicates (n=3). Error bars correspond to the mean and standard deviation.

3.2 List of variants

Table S2. CYP716As wild-type, Variants, and mutations in *in vivo* functional analysis

Sample	Origin	Mutations
CYP716A12_WT	CYP716A12	Wild-type
CYP716A12_D122Q		D122Q
CYP716A12_I212P		I212P
CYP716A12_D292A		D292A
CYP716A12_Q358P		Q358P
CYP716A12_D122Q_Q358P		D122Q_Q358P
CYP716A12_I212P_Q358P		I212P_Q358P
CYP716A15_WT	CYP716A15	Wild-type
CYP716A48_WT	CYP716A48	Wild-type
CYP716A49_WT	CYP716A49	Wild-type
CYP716A1_WT	CYP716A1	Wild-type
CYP716A1_S356Q		S356Q
CYP716A2_WT	CYP716A2	Wild-type
CYP716A2_M206A		M206A
CYP716A2_F210I		F210I

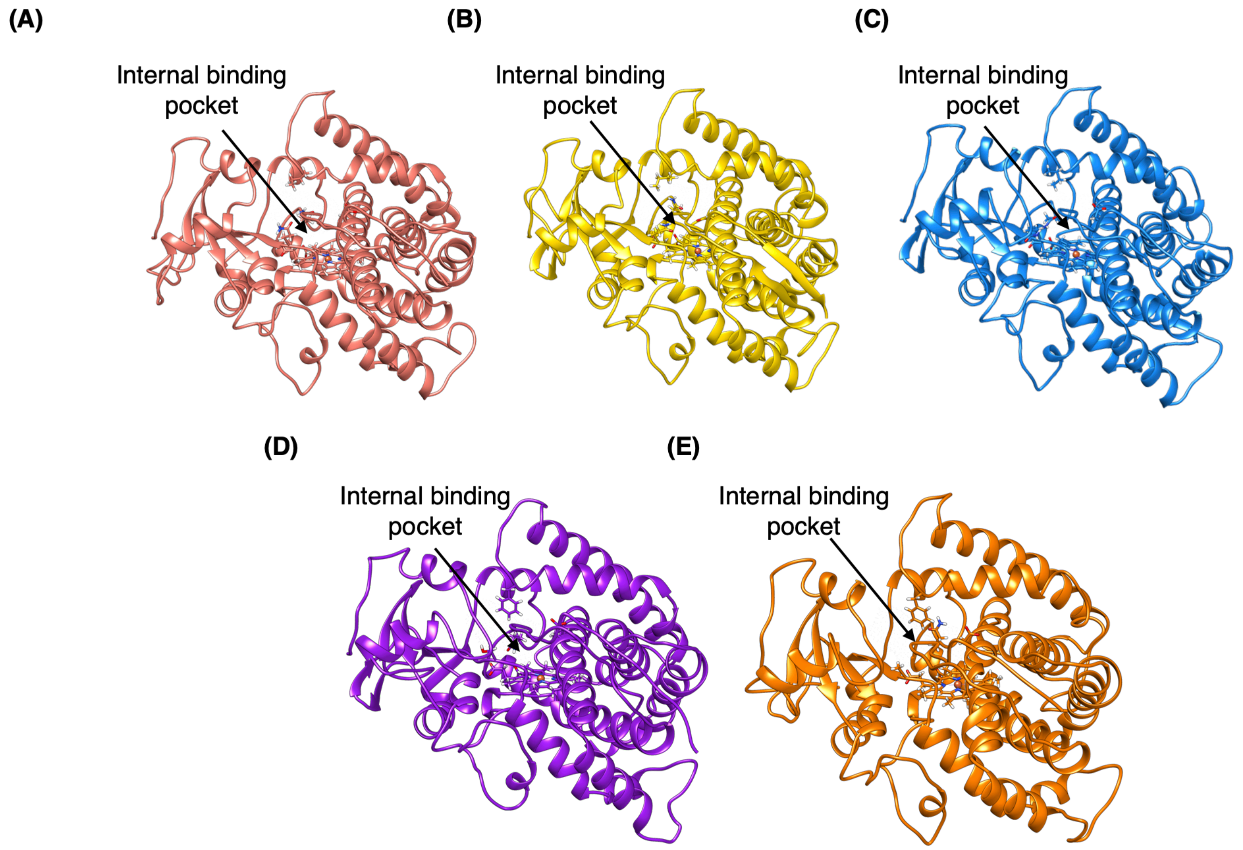

Supplementary Figure 2 Comparative homology modeling of CYP716As with identified internal binding pocket.

The crystal structure of retinoic acid-bound cyanobacterial CYP120A1(2VE3) chain A was selected as a template by homology sequence searching via Protein BLAST. (A) Homology modeling of CYP716A15 was 37.3% identical against the template. (B) Homology modeling of CYP716A48 was 37.8% identical against the template. (C) Homology modeling of CYP716A49 was 36.9% identical against the template. (D) Homology modeling of CYP716A1 was 36.2% identical against the template. (E) Homology modeling of CYP716A2 was 36.6% identical against the template. All homology modeling was passed to pass quality criteria verified by VERIFY3D, with at least 80% of the amino acids having scored >= 0.2 in the 3D/1D profile.

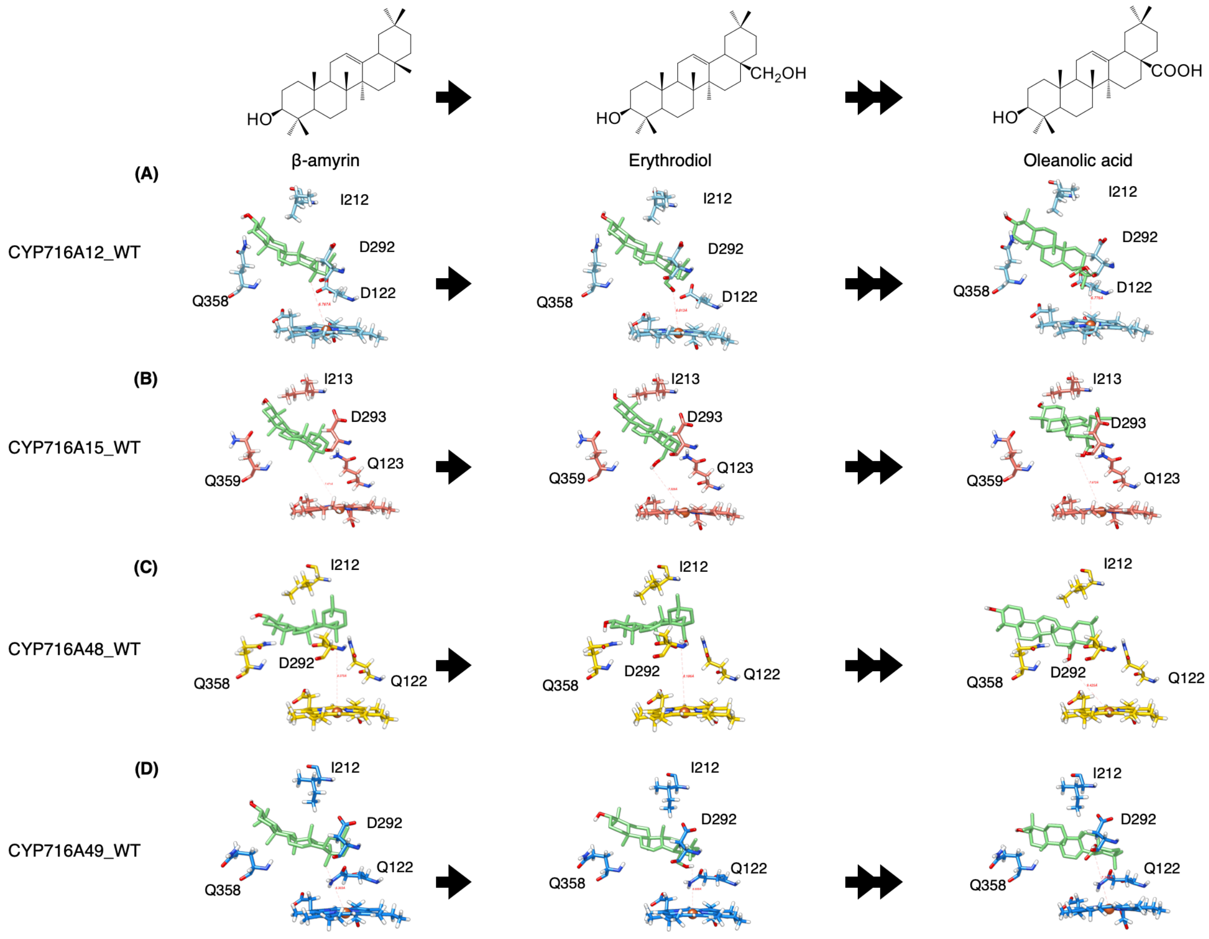

Supplementary Figure 3 Representative interactions of selected CYP716As against β-amyrin backbone and its derivatives (erythrodiol and oleanolic acids).

The structure homology modeling showed the β-amyrin backbone and its derivatives in wild type of (A) CYP716A12, (B) CYP716A15, (C) CYP716A48, and (D) CYP716A49.

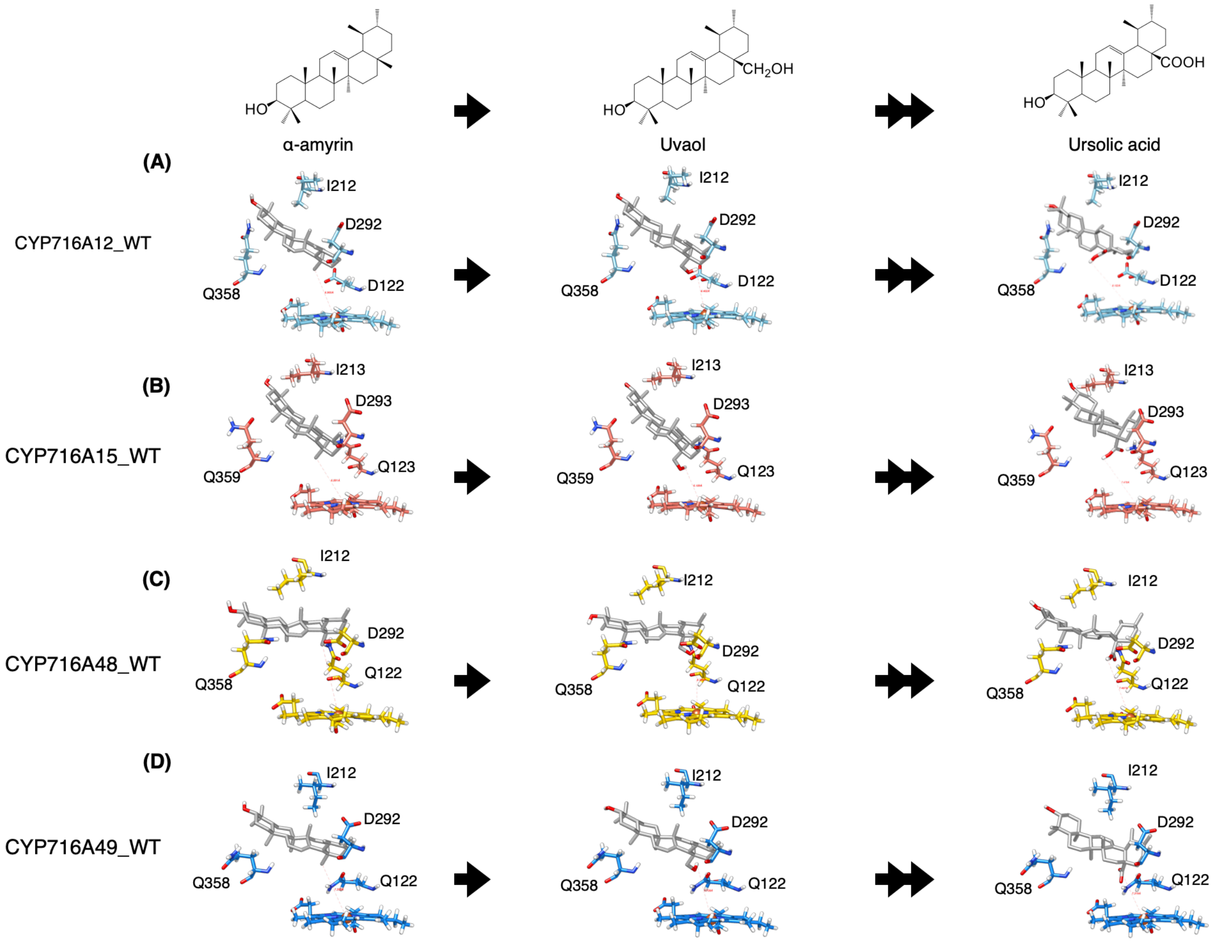

Supplementary Figure 4 Representative interactions of CYP716As against α-amyrin backbone and its derivatives (uvaol, ursolic acid).

The structure homology modeling showed α-amyrin backbone and its derivatives in wild type of (A) CYP716A12, (B) CYP716A15, (C) CYP716A48, and (D) CYP716A49.

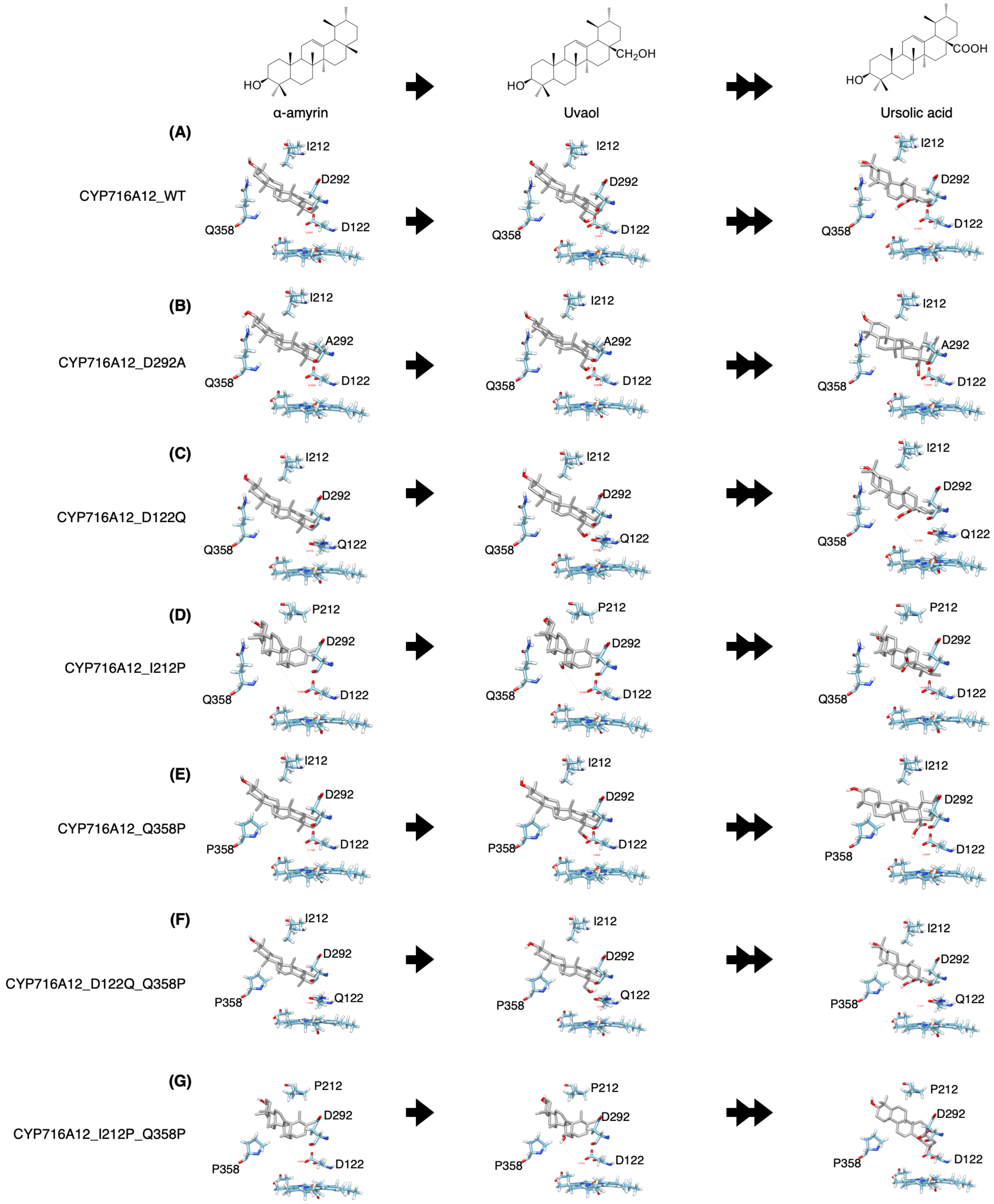

Supplementary Figure 5 Representative interactions of CYP716A12 and its variants against α-amyrin backbone and its derivatives (uvaol, ursolic acid).

The structure homology modeling showed α-amyrin backbone and its derivatives in wild-type of (A) CYP716A12, and CYP716A12 variant (B) CYP716A12_D292A, (C) CYP716A12_D122Q,

(D) CYP716A12_I212P (E) CYP716A12_Q358P, (F) CYP716A12_D122Q_Q358P, and (G) CYP716A12_I212P_Q358P.

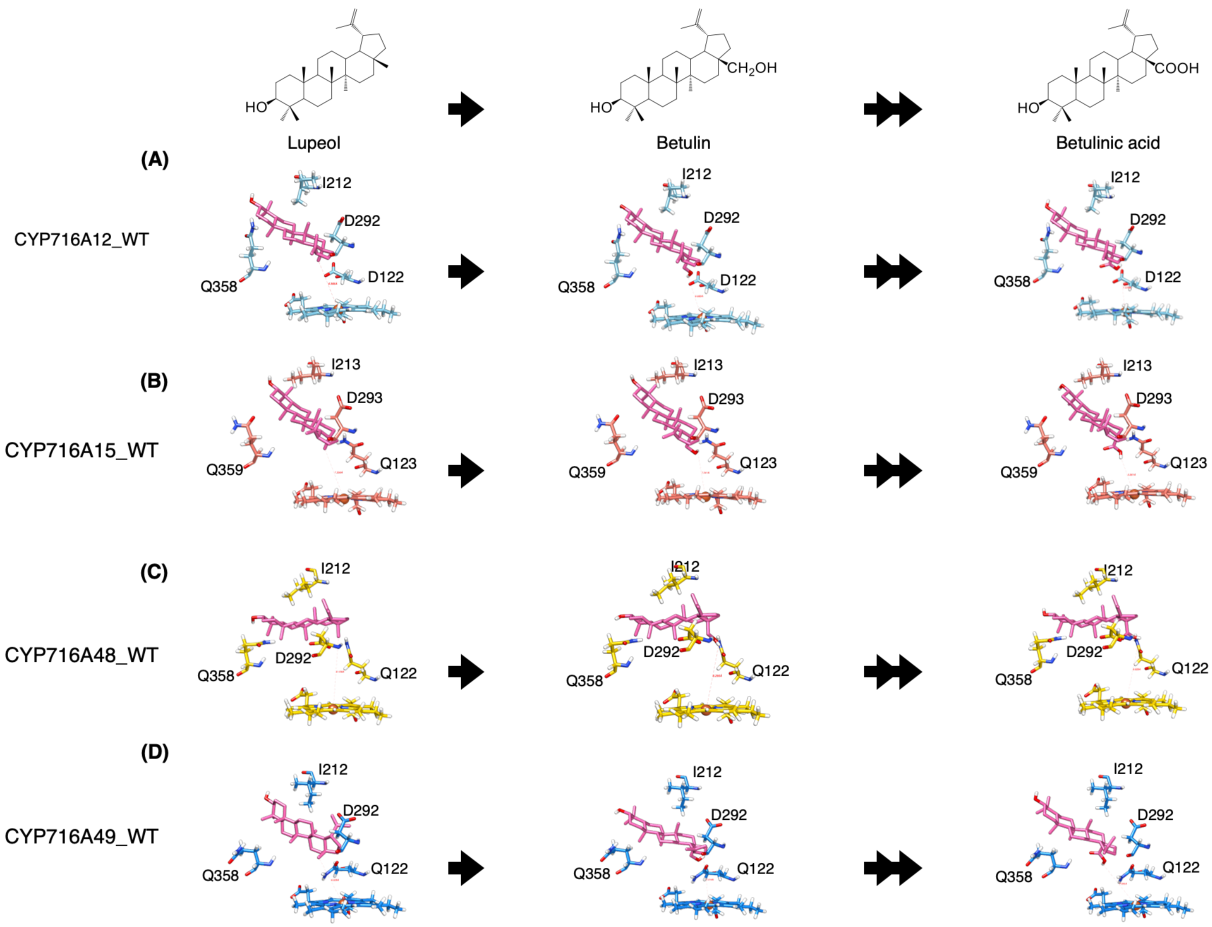

Supplementary Figure 6 Representative interactions of selected CYP716As against lupeol backbone and its derivatives (betulin, betulinic acid).

The structure homology modeling showed the lupeol backbone and its derivatives in wild type of (A) CYP716A12, (B) CYP716A15, (C) CYP716A48, and (D) CYP716A49.

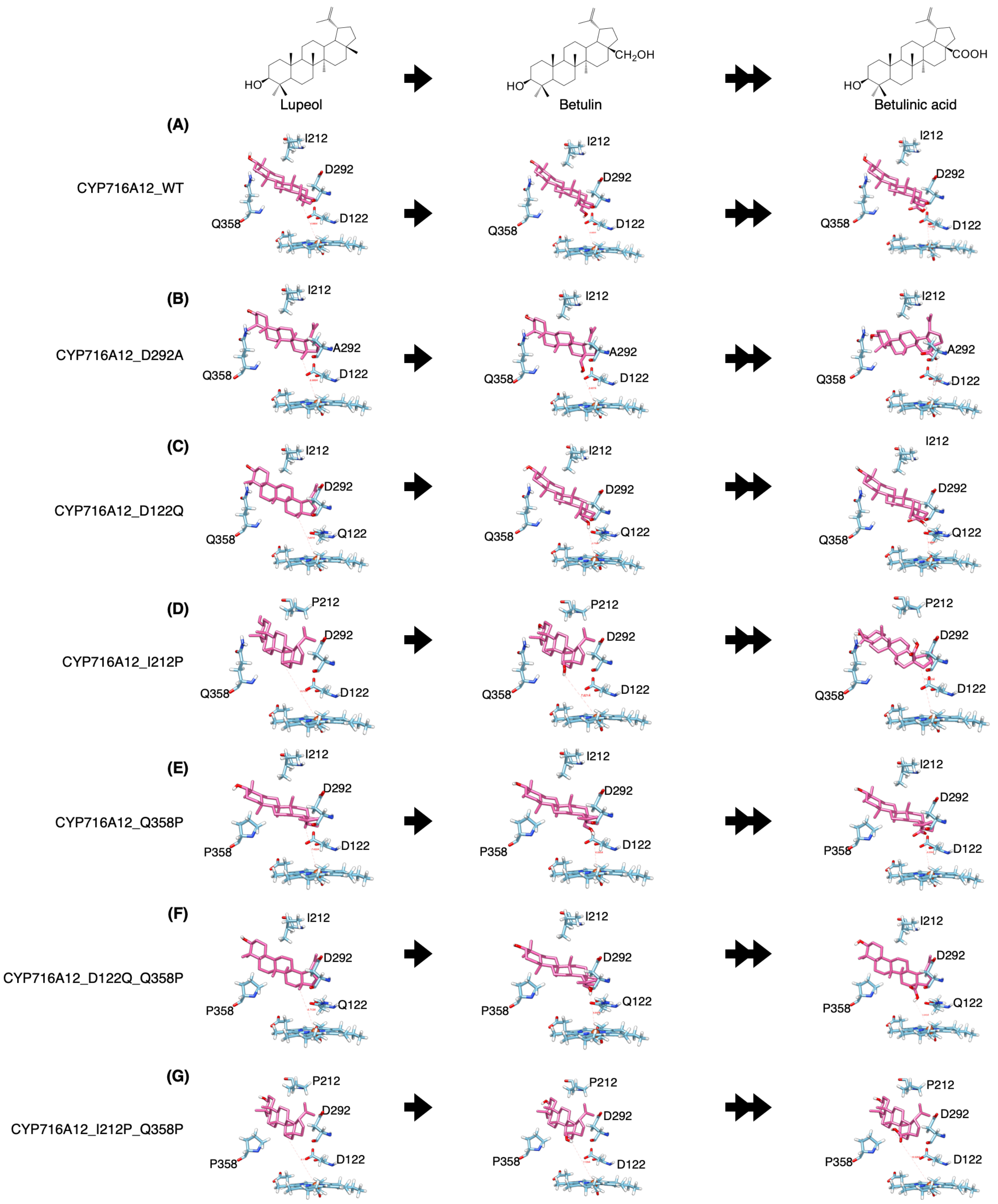

Lupeol
Betulin
Betulinic acid
CH_2OH
COOH
HO
(A)
CYP716A12_WT
(B)
CYP716A12_D292A
(C)
CYP716A12_D122Q
(D)
CYP716A12_I212P
(E)
CYP716A12_Q358P
(F)
CYP716A12_D122Q_Q358P
(G)
CYP716A12_I212P_Q358P
I212
P212
D292
A292
D122
Q122
Q358
P358

Supplementary Figure 7 Representative interactions of CYP716A12 and its variants against lupeol backbone and its derivatives (betulin, betulinic acid).

The structure homology modeling showed lupeol backbone and its derivatives in wild type of (A) CYP716A12, and CYP716A12 variant (B) CYP716A12_D292A, (C) CYP716A12_D122Q, (D) CYP716A12_I212P, (E) CYP716A12_Q358P, (F) CYP716A12_D122Q_Q358P, and (G) CYP716A12_I212P_Q358P

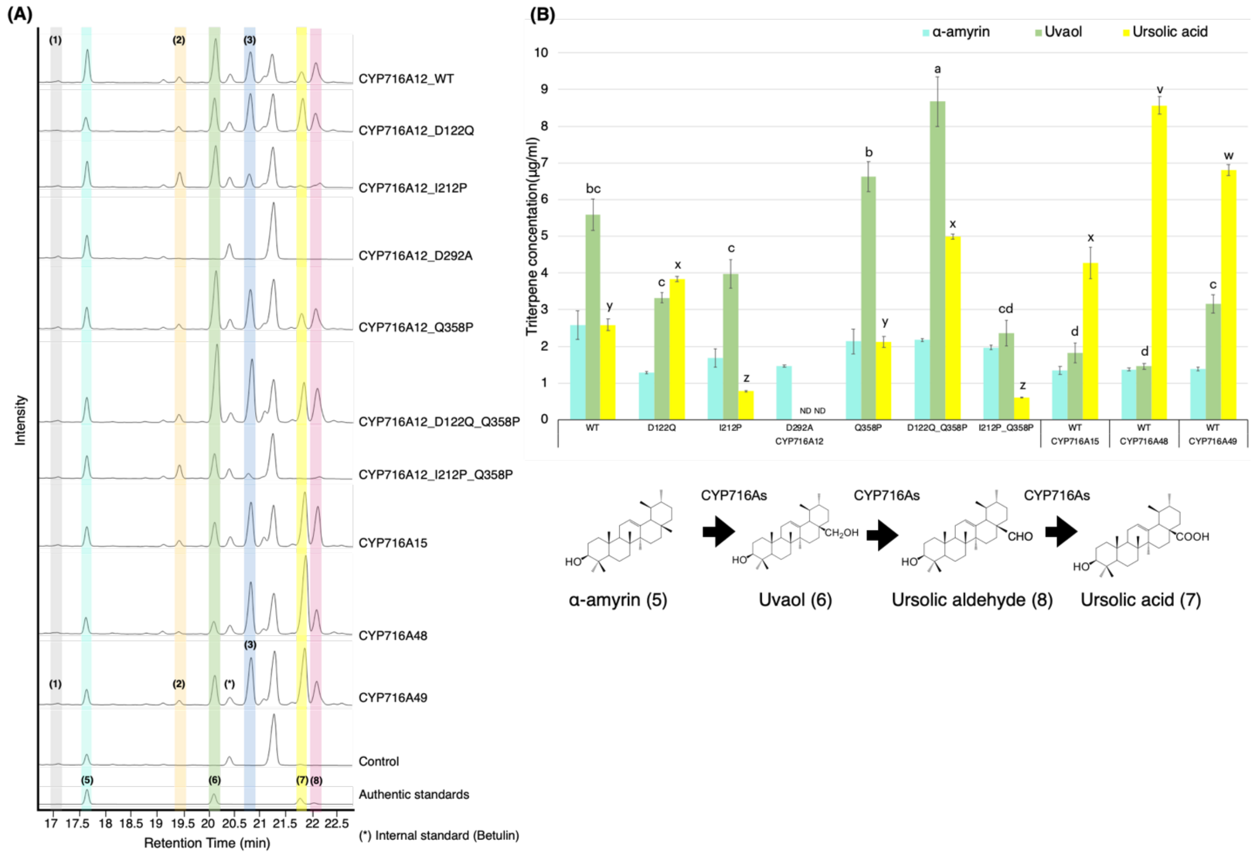

Supplementary Figure 8 *In vivo* activities of selected CYP716As and CYP716A12, and its variants against α-amyrin skeletons.

(A) TICs of extracts from yeast harboring one *aAS* expression vector for producing α-amyrin as a major substrate and three CYPs expression vectors (pELC-CYP716As, pYES-DEST52-CYP716As, and pESC-HIS-CYP716As) were used. (B) Quantification of triterpene concentration in yeast harboring CYP716As against α-amyrin skeletons. Quantitation and error bars correspond to the mean and standard deviation, respectively. Data are representative of at least three biological replicates (n=3). Letters indicate statistical differences between the different oxidized triterpenoids levels (a-d: uvaol levels, v-z: ursolic acid levels) for each sample (one-way ANOVA; Tukey's post-hoc test, $p \leq 0.05$). ND, not detected.

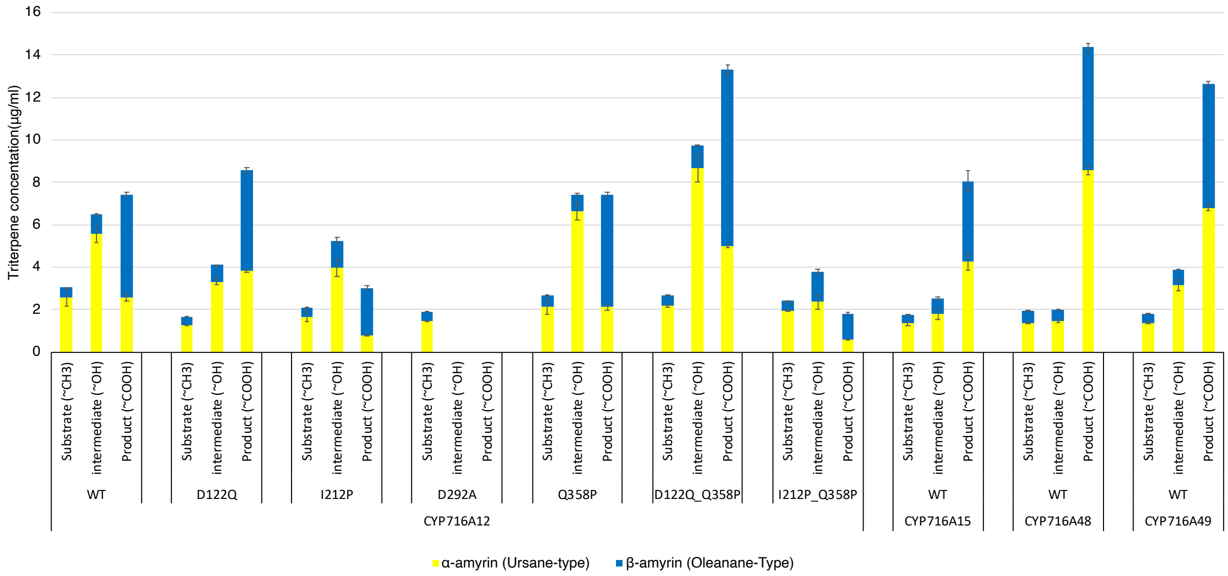

Supplementary Figure 9 Comparison of triterpene concentration from extracts of yeast producing α-amyrin (Ursane-type) as a major substrate and β-amyrin (Oleanane-Type) as a minor substrate.

Yeast harboring one *aAS* expression vector for making α-amyrin as a major substrate and three CYPs expression vectors (pELC-CYP716A, pYES-DEST52-CYP716As, and pESC-HIS-CYP716As) were used. Quantitation and error bars correspond to the mean and standard deviation, respectively. Data are representative of at least three biological replicates (n=3). ND, not detected.

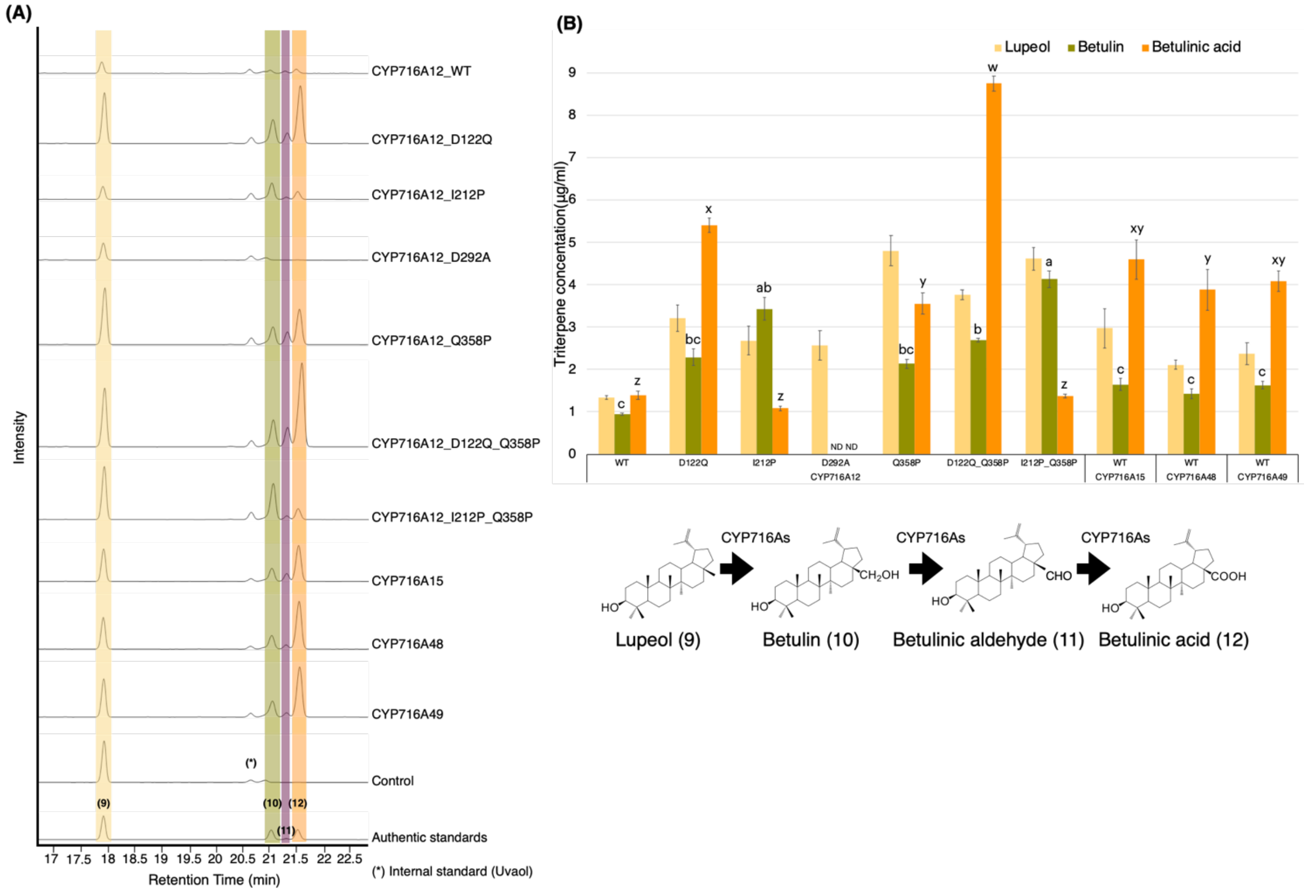

Supplementary Figure 10 *In vivo* activities of selected CYP716As and CYP716A12, and its variants against lupeol skeletons.

(A) Extracted ion chromatogram (EIC) by selecting *m*/*z* of extracts from yeast harboring one *LUS* expression vector for producing lupeol, and three CYP harbouring expression vectors (pELC-CYP716As, pYES-DEST52-CYP716A, and pESC-HIS-CYP716A) were used. (B) Quantification of triterpene concentration in yeast harboring CYP716As against lupeol skeletons. Quantitation and error bars correspond to the mean and standard deviation, respectively. Data are representative of at least three biological replicates (n=3). Letters indicate statistical differences between the different oxidized triterpenoids levels (a-c: betulin levels, w-z: betulinic levels) for each sample (one-way ANOVA; Tukey's post-hoc test, $p \leq 0.05$). ND, not detected.
